# Structural and functional insights into the helicase protein E5 of Mpox virus

**DOI:** 10.1038/s41421-024-00680-1

**Published:** 2024-06-25

**Authors:** Weizhen Zhang, Yusong Liu, Mengquan Yang, Jie Yang, Zhiwei Shao, Yanqing Gao, Xinran Jiang, Ruixue Cui, Yixi Zhang, Xin Zhao, Qiyuan Shao, Chulei Cao, Huili Li, Linxi Li, Hehua Liu, Haishan Gao, Jianhua Gan

**Affiliations:** 1 Shanghai Sci-Tech Inno Center for Infection & Immunity, State Key Laboratory of Genetic Engineering, Collaborative Innovation Center of Genetics and Development, Department of Biochemistry and Biophysics, School of Life Sciences, Fudan University, Shanghai, China; 2 School of Life Sciences, Westlake University, Hangzhou, Zhejiang China; 3 Westlake Laboratory of Life Sciences and Biomedicine, Hangzhou, Zhejiang China; 4 Department of Geriatrics, Medical center on Aging of Shanghai Ruijin Hospital, Shanghai Jiaotong University school of Medicine, Shanghai, China

**Keywords:** Cryoelectron microscopy, X-ray crystallography

## Abstract

Mpox virus (MPXV) can cause mpox in humans. Due to its quick and wide spread in the past two years, mpox has turned into a significant public health concern. Helicase E5 is a multi-domain protein; its primer synthesis and DNA unwinding activity are required for genome uncoating and DNA replication of MPXV. However, the in vitro DNA unwinding activity has never been demonstrated. Here, we report the structural and biochemical studies of MPXV E5, showing that the full-length protein adopts an auto-inhibited conformation. Truncation of the N-terminus can recover the in vitro unwinding activity of E5 towards the forked DNA. Further structural analysis reveals that MPXV E5 shares a conserved mechanism in DNA unwinding and primer synthesis with the homologous proteins. These findings not only advance our understanding on the function of MPXV E5, but also provide a solid basis for the development of anti-poxvirus drugs.

## Introduction

MPXV belongs to the genus orthopoxvirus within the Poxviridae family. Although it was originally identified from monkeys in 1958^1^, later studies showed that many rodents, such as mice, rats, and squirrels, are also the natural reservoir hosts for MPXV^2^. Similar to Variola virus (VARV, the causative agent of smallpox), Vaccinia virus (VACV), Cowpox virus (CPXV), and all other members within the orthopoxvirus genus, MPXV can be transmitted from animals to humans, leading to mpox; the clinic symptoms of human mpox resemble that of discrete smallpox^3,4^. MPXV possesses four main strains. Among them, the SL-V70, COP-58, and WRAIR-6 strains are grouped into the West African clade (or Clade I) with a mortality rate of 3.6%. The ZAI-96 strain belongs to the Central African clade (or Clade II)^2,5^. Owing to the presence of some extra genes involved in apoptotic regulation, the Clade II MPXV is more pathogenic and could lead to up to 10% of death^6,7^.

Unlike smallpox, the human-to-human transmission rate of previous mpox is low, which restrained mpox within the African continent till 2003^8^. However, since May 2022, human mpox has been widely spread^9–11^. As of 9th August 2023, more than 89,000 total cases and 150 deaths were reported in overall 150 countries, which turned MPXV into a significant public health concern. The 2022-3 mpox outbreak is caused by the strains belonging to the less transmissible Clade I^12^; however, its clinical and epidemiological features are remarkably different from previous outbreaks. The detailed causes of the change in epidemiology, spread and clinical symptoms of mpox remain elusive, but sequence analysis has confirmed that genes essential for the replication process are highly conserved in MPXV^12^ and all other orthopoxvirus members.

MPXV is an enveloped, double-stranded DNA virus; the genome is about 197,000 kb in size and encodes more than 190 proteins^13,14^. Upon entry into the host cells, the genome of MPXV is rapidly liberated through uncoating and replicated in the cytoplasm. As demonstrated by VACV, the prototypical and best-studied member of orthopoxvirus, all proteins directly involved in and essential for viral DNA replication are encoded by their own genome^15–17^. In addition to DNA polymerase E9^18^, processivity factor A20^19^, uracil DNA glycosidase D4^19^, phosphoprotein H5^20^, and the single-stranded DNA-binding protein I3^21^, the helicase protein D5 also plays very critical roles in viral DNA replication of VACV^20^. D5 is a multi-domain protein^22^. Besides the helicase domain at the C-terminus, it also contains an archaeoeukaryotic primase (AEP) domain at its N-terminus, which plays a key role in initiating DNA replication^23^. In addition to replication, a previous study showed that the helicase activity of D5 is also required for in vivo uncoating of the viral genome^24^. However, the helicase activity has not been demonstrated in vitro for VACA D5 or the homologs from any other orthopoxvirus members.

MPXV proteins A22, E4, E5, F9, H5, and I3 are the homologs of A20, D4, D5, F8, H5, and I3 in VACA, respectively. Recently, several cryo-EM structures have been reported, revealing the detailed conformation and assembly of A22, E4, F9, and H5 in the DNA replication machine of MPXV^25–28^. However, the structural information of MPXV E5 and the homologous proteins is very limited. To date, only one low-resolution cryo-EM structure is available for VACV D5, in which the AEP domain was truncated^29^. Here, we report extensive structural and biochemical studies of MPXV E5. Our structure shows that the full-length MPXV E5 forms an asymmetric, auto-inhibited hexamer. The AEP domain from one monomer sits right next to the entrance, preventing DNA from entering the central channel in the correct orientation. Truncation of the N-terminus can recover the DNA unwinding activity of MPXV E5 in vitro. Instead of DNA with either 5′ or 3′-overhang, MPXV E5 preferentially unwinds forked DNA. Moreover, we also identified many critical residues for the DNA unwinding and primer synthesis activities of MPXV E5. Our studies not only advance our understanding on the function of the E5 protein, but also provide a solid basis for the development of anti-poxvirus drugs.

## Results

### Full-length MPXV E5 adopts an asymmetric conformation

**Fig. 1:**
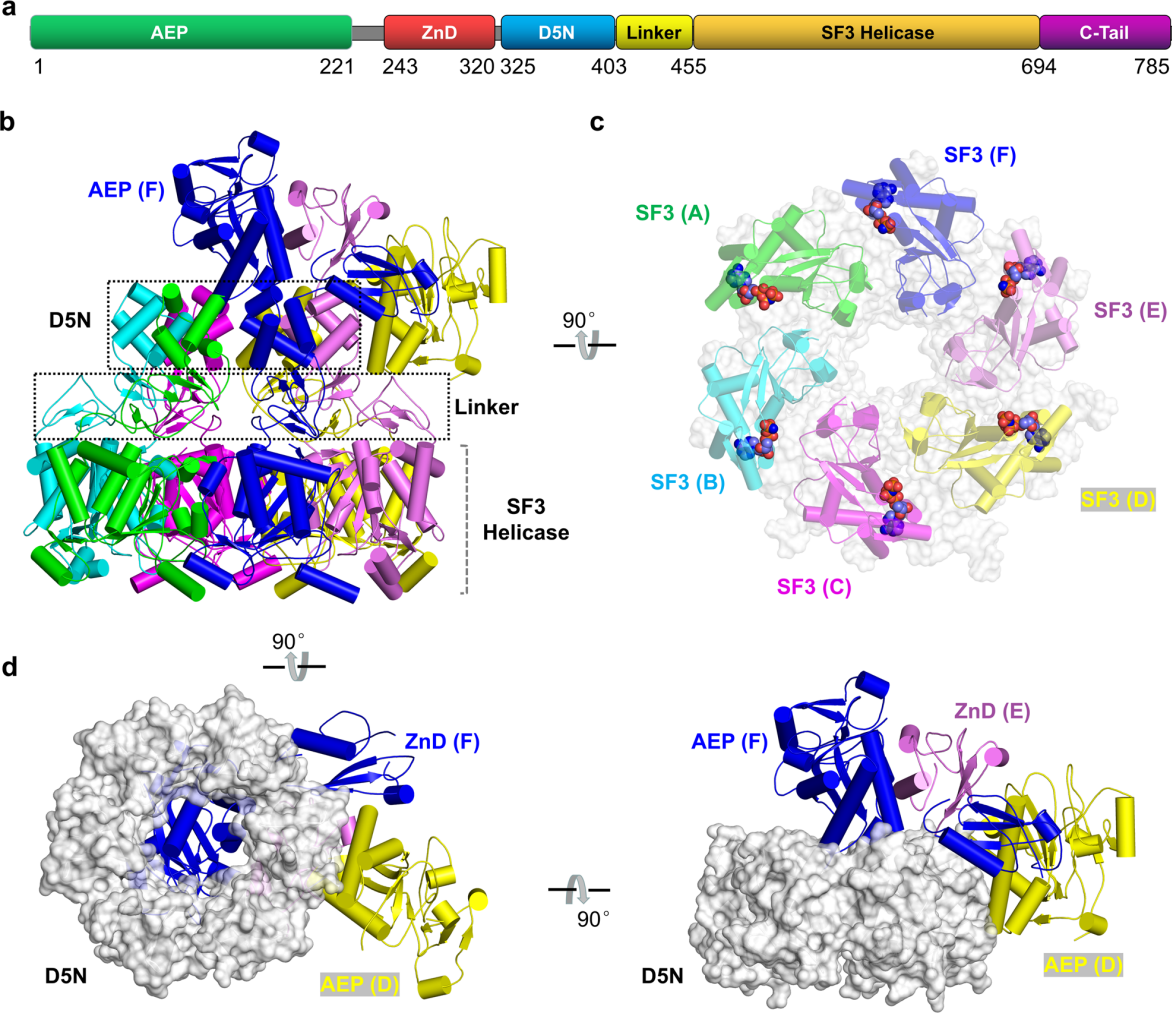
Cryo-EM structure of MPXV E5-AMPPNP complex. **a** Domain architecture of MPXV E5. **b** Overall folding and assembly of MPXV E5 in the E5-AMPPNP structure. **c** The central ring formed by the D5N, Linker, and SF3 helicase domains, which are shown as cartoon in different colors. D5N and SF3 domains are shown as surface in gray. The bound AMPPNPs are shown as spheres. **d** The relative orientations of the AEP and ZnD domains, which are shown as cartoon. D5N domains are shown as surface in gray.

MPXV E5 is encoded by the *E5R* gene, the mature protein is 785 amino acids (aa) in length. MPXV E5 shares a very high sequence identity with the homologous proteins (Supplementary Fig. S1), suggesting that they may possess similar structures and biological functions. MPXV E5 can be divided into 6 domains: the AEP domain, the ZnD domain (Zinc-binding domain), the D5N domain (a domain associated with D5-type helicases), the Linker, the SF3 Helicase domain, and the C-Tail (Fig. 1a). To better understand its function, we expressed and purified the full-length MPXV E5 protein. Using DNAs with 5′ and/or 3′ overhangs as substrates (Supplementary Fig. S2a and Table S1), in vitro DNA unwinding assays were performed. As depicted in Supplementary Fig. S2b, the full-length MPXV E5 shows no detectable unwinding activity toward any of the tested DNAs. Like MPXV E5, no helicase activity has been observed for the helicases of VACV and other orthopoxviruses^22^.

Although it could not unwind DNA, previous studies confirmed that VACV D5 possesses strong in vitro ATPase activity and DNA binding ability^22,30^. Puzzled by these observations, we performed cryo-EM studies (Supplementary Fig. S3) for MPXV E5 in the presence of AMPPNP and forked DNA, which has overhangs at both 5′ and 3′ ends (Supplementary Table S1). One E5-AMPPNP complex structure (Supplementary Figs. S4-S5) was determined with an overall resolution of 3.32 Å (Supplementary Table S2). As shown in the structure (Fig. 1b), MPXV E5 assembles into hexamer. The central D5N, Linker and SF3 helicase domains are well-ordered, forming a ring-like conformation (Fig. 1c). The orientations between the three domains are relatively conserved in the monomers A, B, C, and F (Supplementary Fig. S6a). However, when aligned based on the D5N domain, the relative orientations of the SF3 domains of the monomers D and E are different from other monomers (Supplementary Fig. S6b). Due to the weak electron densities, no C-Tail could be built in the model.

Two AEP (from monomers D and F) and two ZnD (from monomers E and F) domains are well-ordered in the structure. Owing to their poor electron densities, other AEP and ZnD domains are not built. As indicated by the low root-mean-square deviation value (RMSD, 0.7 Å), the overall foldings of the two AEP domains are very similar (Supplementary Fig. S7a); however, they adopt dramatically different orientations (Fig. 1d). The AEP domain of monomer D resides next to the D5N domain of monomer E, and is flanked by the ZnD domains of monomers E and F. The AEP domain of monomer F sits right on top of the ring formed by the D5N domains (Fig. 1d). Compared to monomer D, the AEP domain undergoes approximately 90° rotations in monomer F (Supplementary Fig. S7b). The ZnD domain is composed of one short α-helix and three anti-parallel β-strands (Supplementary Fig. S7c); the Zn^2+^ ion is coordinated with the side chains of Cys282, Cys285, His290, and Cys314 (Supplementary Fig. S7d). The relative orientations of the two ZnD domains are very different (Supplementary Fig. S7e). Altogether, these observations suggested that the full-length MPXV E5 protein adopts an asymmetric conformation in vitro.

### MPXV E5 can bind ssDNA

**Fig. 2:**
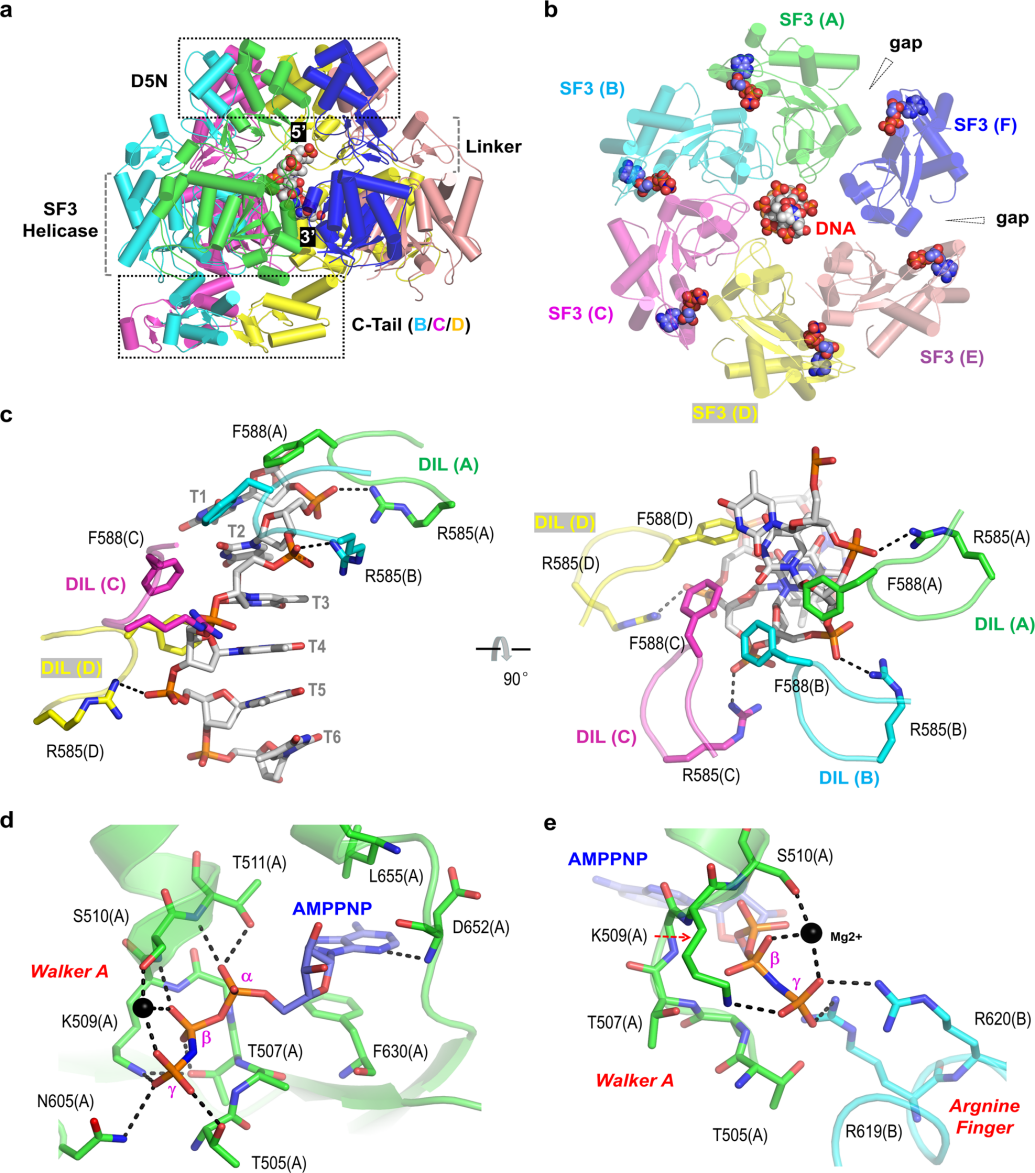
DNA and AMPPNP recognition in the E5-ssDNA-AMPPNP complex. **a** Overall folding and assembly of MPXV E5 in the complex structure. **b** Conformations of DNA and SF3 helicase domains in the complex structure. DNA and the bound AMPPNP are shown as spheres. **c** The detailed interactions between DNA and residues from the DIL loops of SF3 helicase domains. **d**, **e** AMPPNP recognition by the SF3 helicase domains. Mg^2+^ ion is shown as sphere in black.

Besides the E5-AMPPNP complex, one E5-ssDNA-AMPPNP complex structure (Supplementary Fig. S8a) was also determined with an overall resolution of 2.74 Å (Supplementary Table S2). Compared to the nucleobases, the electron densities for the phosphate backbones are better defined (Supplementary Fig. S8b), confirming that it is the ssDNA region captured in the structure. Totally, six nucleotides (nt) could be built, which all reside in the central channel formed by the D5N, Linker, and SF3 helicase domains (Fig. 2a). The 5′-end of the DNA points toward the D5N domains, whereas the 3′-end points towards the opposite side of the ring.

The orientations of the D5N and Linker domains are relatively conserved, but the SF3 helicase domains adopt various orientations in the E5-ssDNA-AMPPNP complex. As depicted in Fig. 2b, the SF3 helicase domains from monomers A to E are next to each other, whereas the one from monomer F is relatively isolated. When aligned based on the D5N domains, the SF3 helicase domains of monomers A to D undergo sequential titling; compared to monomer A, the shifting of the SF3 helicase domain can be up to 11-Å in monomer D (Supplementary Fig. S9a). The SF3 helicase domains from monomers A to D are arranged into a spiral-like configuration, placing their DNA-interacting loop (DIL loops, aa 580–595) towards the bound DNA (Fig. 2c). The conformation of the DNA is mainly stabilized by residues Arg585 and Phe588 from the DIL loops. The side chains of Arg585 form hydrogen bond (H-bond) interactions with the backbone phosphate groups of the DNA; as indicated by the average distance (2.8 Å), these H-bonds are very stable. The side chain six-member rings of Phe588 pack against the sugar puckers of the DNA.

In addition to the tilting of the whole domain (Supplementary Fig. S9a), direct superposition indicated that the SF3 helicase domains also undergo obvious local conformational changes, especially in the DIL loop regions (Supplementary Fig. S9b). Owing to interaction with the DIL loop, the conformation of another loop (L1 loop, aa 535–542) also varies from one monomer to another. The SF3 helicase domains of monomers E and F do not interact with the DNA (Fig. 2b, c), their DIL and L1 loops are completely disordered (Supplementary Fig. S9c). No DIL and L1 loop could be observed in the E5-AMPPNP structure. Taken together, these observations suggested that the local conformational changes are associated with DNA binding to some extent.

### ATP recognition by the SF3 helicase domain of MPXV E5

The DNA unwinding activity has not been demonstrated for MPXV E5 and the homologous proteins, but previous studies have confirmed that VACV D5 can bind and hydrolyze ATP (or other NTP)^22,30^. To unravel the structural basis for ATP binding and hydrolysis, one non-hydrolyzable ATP analog, AMPPNP, was utilized in the cryo-EM sample preparation. In the E5-ssDNA-AMPPNP structure, six AMPPNP molecules were captured, and each was associated with one SF3 helicase domain (Fig. 2b; Supplementary Fig. S10a). The conformation of AMPPNP is stabilized by various types of interactions (Fig. 2d). Besides the H-bond interaction with the main chain N atom of Asp652, the nucleobase of AMPPNP also forms extensive stacking interaction between the side chains of Phe630 and Leu655. The α-phosphate forms two H-bond interactions with Thr511, one with the main N atom and the other with the side chain OG1 atom. The β-phosphate also forms two H-bond interactions, between its O1B and O2B atoms and the side chain OG1 atom of Thr507 and the main chain N atom of Ser510, respectively. The γ-phosphate interacts with more residues from the same monomer, such as Thr505, Lys509, and Asn605. In addition, the γ-phosphate also forms H-bond interactions with two Arg residues (Arg619 and Arg620) from the adjacent monomer (Fig. 2e). The conformation of AMPPNP is further stabilized by coordination with one Mg^2+^ ion (Supplementary Fig. S8c), which was also included in cryo-EM sample. The Mg^2+^ ion coordinates with the β- and γ-phosphate groups and the side chain OG atom of Ser510 (Fig. 2d, e). Our structural observations are consistent with a previous study on VACV D5, which showed that the N-terminal region (aa 1–300) is dispensable, but oligomerization and participation of several key residues (including Lys509, Arg619, and Arg620) are required for ATP hydrolysis^31^.

The detailed conformations and interactions are shared by AMPPNPs bound by the monomers A to D, whereas the γ-phosphate groups are disordered for the AMPPNPs associated with the monomers E and F. Likely, due to the lack of interaction between AMPPNP γ-phosphate and Arg619 and Arg620 from the adjacent monomers, the SF3 helicase domain of monomer F forms two big gaps in the structure (one with monomer A and the other with monomer E, Fig. 2b). Six AMPPNP molecules were also captured in the apo-form MPXV E5 structure (Fig. 1c); however, none of them forms stable interaction with either Arg619 or Arg620 from the adjacent monomers.

### Comparison of the DNA and ATP binding modes with other SF3 members

**Fig. 3:**
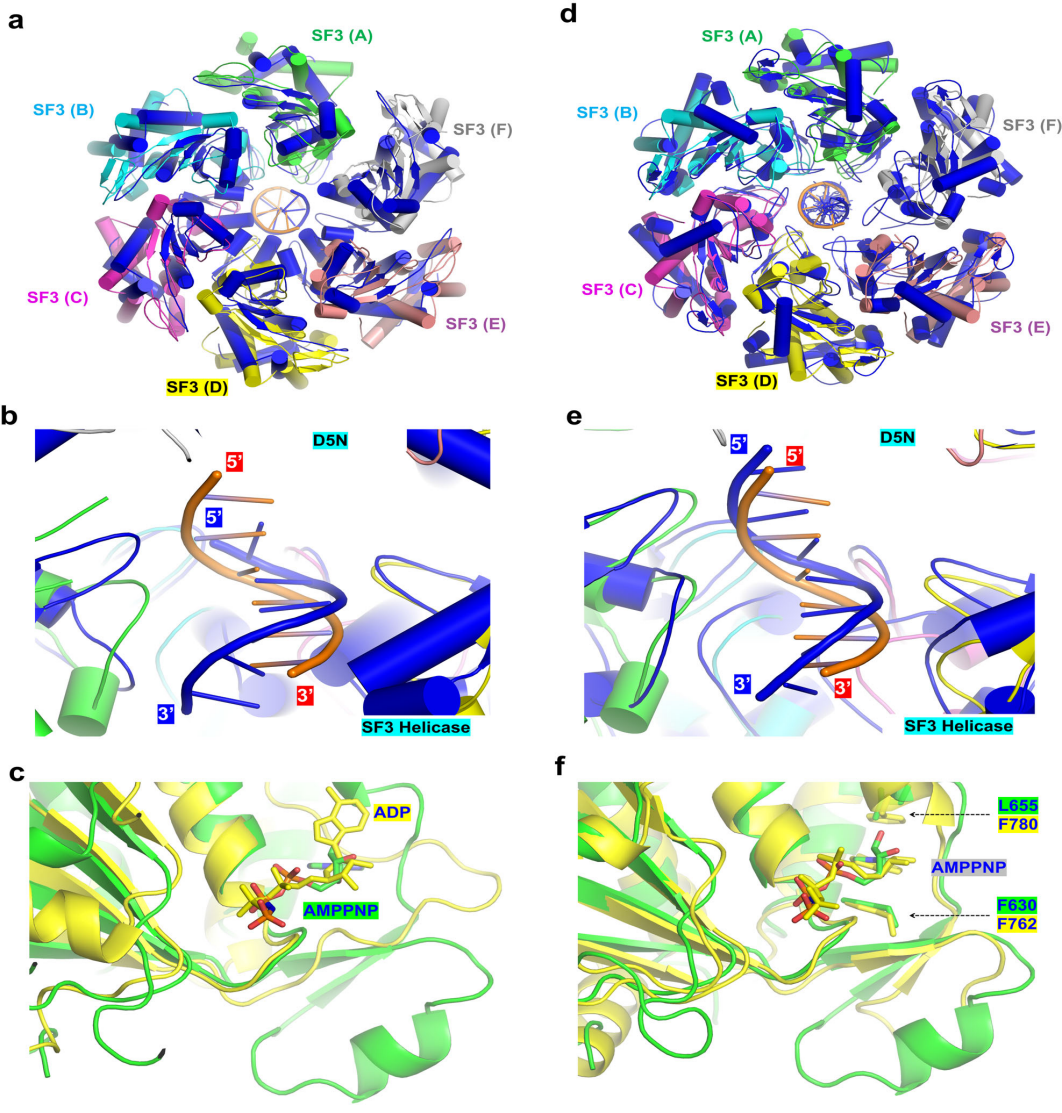
Structural comparison between MPXV E5 and other SF3 superfamily proteins. **a**–**c** Superposition of the E5-ssDNA-AMPPNP complex and the HPV E1-DNA complex. HPV E1 and the bound DNA are colored in blue in **a** and **b**. In **c**, HPV E1 and the bound ADP are colored in yellow. **d**, **e** Superposition of the E5-ssDNA-AMPPNP complex and the ASFV C962R-DNA complex. ASFV C962R and the bound DNA are colored in blue in **d** and **e**. In **f**, ASFV C962R and the bound AMPPNP are colored in yellow.

MPXV E5 assembles into ring-shaped hexamer in both the E5-AMPPNP and E5-ssDNA-AMPPNP structures (Figs. 1c and 2b). Hexameric assembly has been observed for the RecA family and SF3 family helicases, which unwind DNA from the 5′-end and 3′-end, respectively^32^. As depicted in Supplementary Fig. S11, the helicase domain of MPXV E5 shares sequence similarity with many known SF3 family members, such as the C962R protein of African swine fever virus (ASFV)^33^, the E1 helicase of human papillomavirus (HPV)^34^, and the PrimPol protein of Nitratiruptor phage NrS-1^35^. No DNA-bound structure is available for NrS-1 PrimPol, whereas they have been reported for HPV E1 (PDB_ID: 2GXA)^34^ and ASFV C962R (PDB_ID: 8IQI)^33^. In the E1 structure, the DNA is mainly stabilized by one Lys residue and one His residue, which are next to each other in sequence. In both MPXV E5 and C962R structures, the DNAs are stabilized by one Arg residue and one additional residue; the two DNA-interacting residues are separated by two amino acids in the middle. Although the DNA-interacting residues are not absolutely conserved, the conformations of the bound ssDNAs are very similar in the MPXV E5, HPV E1 (Fig. 3a, b), and ASFV C962R structures (Fig. 3d, e).

The in vitro DNA unwinding activities of NrS-1 PrimPol^35^, HPV E1 and ASFV C962R^33^ have been previously reported and confirmed that ATP (or NTP) binding and hydrolysis are required for DNA unwinding. The majority of the ATP-binding residues of MPXV E5 are conserved and located at the Walker A, Sensor 1, and Arginine Finger regions (Supplementary Fig. S11), which are the characteristic motifs of SF3 helicases^32^. Structural superposition showed that the conformations of the nucleobases are different, but the phosphate groups adopt similar conformations in the MPXV E5 and HPV E1 structures (Fig. 3c). Like the MPXV E5 structure, AMPPNP was also captured in the ASFV C962R structure, and the conformations of the AMPPNPs are virtually identical in the two structures (Fig. 3f). Interestingly, the nucleobase of AMPPNP is also flanked by two hydrophobic residues (Phe762 and Phe780) in the ASFV C962R structure^33^.

### In vitro DNA unwinding activity is inhibited by the N-terminal domains

**Fig. 4:**
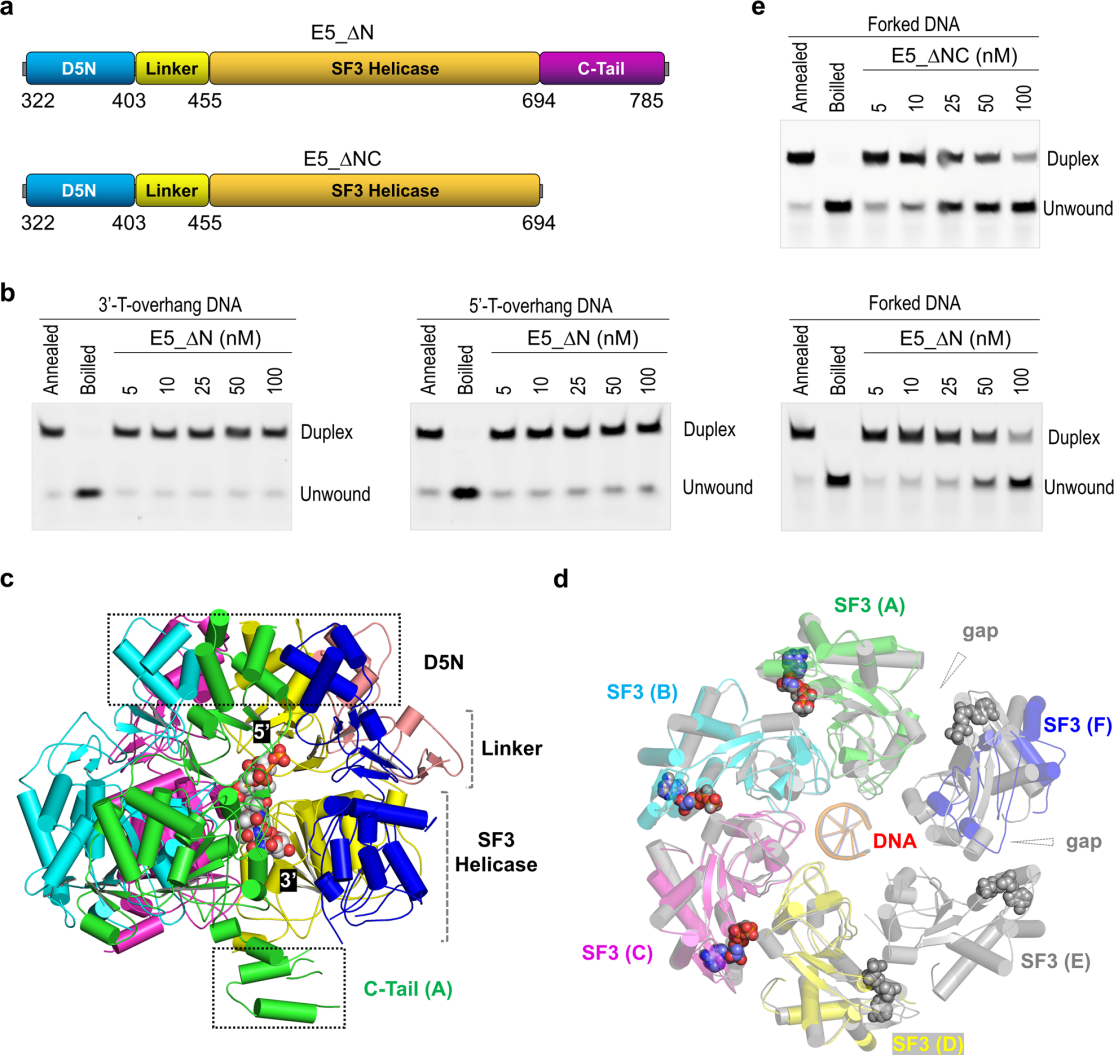
DNA unwinding by the truncated MPXV E5 proteins. **a** Domain architectures of the truncated MPXV E5 proteins. **b** In vitro DNA unwinding assays catalyzed by the N-terminal truncated MPXV E5 (E5_∆N). **c** The overall folding and assembly of the E5_∆N-ssDNA-AMPPNP complex. The bound DNA is shown as spheres. **d** Superposition of DNA and the SF3 helicase domains in the E5_∆ N-ssDNA-AMPPNP complex and the E5-ssDNA-AMPPNP structure. MPXV E5, and the bound AMPPNP are all colored in gray in the MPXV E5/DNA structure. **e** In vitro DNA unwinding assays catalyzed by MPXV E5 with both N- and C-terminal truncation (E5_∆NC).

The sequence and structural similarities all suggested that the SF3-helicase domain of MPXV E5 is functional; however, no such activity has been observed for MPXV E5, VACV D5, or helicases from other orthopoxviruses in previous in vitro studies^22^. Based on the E5-AMPPNP structure, we suspected that the DNA unwinding activity of MPXV E5 is inhibited by the AEP domain residing at the top of the central ring (Fig. 1d). To this end, we constructed and purified one MPXV E5 protein (aa 322–785, Fig. 4a) with N-terminus truncated, which is termed as E5_∆N hereafter. As shown by in vitro DNA unwinding assays (Fig. 4b), E5_∆N has no unwinding activity towards DNA (50 nM) with T15 at either 5′- or 3′-overhang region (Supplementary Table S1), but the forked DNA can be efficiently unwound by E5_∆N. About 60% of forked DNA is unwound by E5_∆N with a concentration of 50 nM. In the presence of 100 nM E5_∆N, more than 75% of forked DNA is unwound. The genomes of orthopoxviruses are AT-rich in their termini^17^. To investigate whether the sequences affect DNA unwinding by MPXV E5, we synthesized several DNAs with either A15, C15, or G15 at the 5′ or 3′-overhang regions (Supplementary Table S1). As depicted in Supplementary Fig. S12, none of these DNAs can be unwound by E5_∆N. All together, these DNA unwinding assay results indicated that the unwinding activity of MPXV E5 is auto-inhibited by its N-terminal domains; and, instead of sequence, the structure of DNA is more critical for DNA unwinding by MPXV E5.

Encouraged by the above DNA unwinding assay results, we then performed cryo-EM study for E5_∆N in the presence of forked DNA and AMPPNP (Supplementary Fig. S13). One E5_∆N-ssDNA-AMPPNP complex structure (Fig. 4c; Supplementary S14a). Like the E5-ssDNA-AMPPNP complex, the 6-nt ssDNA fragment was captured by the SF3 helicase domains of monomers A to D in the E5_∆N-ssDNA-AMPPNP structure. Structural superposition shows that the conformations of the DNA and the four DNA-interacting SF3 helicase domains are virtually identical in the two complex structures (Fig. 4d), indicating that truncation of the N-terminus does not alter the DNA-binding mode by MPXV E5. Compared to the E5-ssDNA-AMPPNP structure, the overall resolution of the E5_∆N-ssDNA-AMPPNP structure is higher (2.67 Å, Supplementary Table S2). As a result, better-defined electron density maps were observed for the bound AMPPNP (Supplementary Fig. S10b), ssDNA (Supplementary Fig. S14b), and residues important for AMPPNP or ssDNA binding (Supplementary Fig. S15).

Three AMPPNP molecules were captured in the E5_∆N-ssDNA-AMPPNP structure (Supplementary Fig. S14c, d), associated with the SF3 domains of monomers A, B, and C, respectively; the conformations of the AMPPNPs are identical to these in the E5-ssDNA-AMPPNP structure (Fig. 4d). No AMPPNP was associated with the monomers D and F in the E5_∆N-ssDNA-AMPPNP structure; their ATP-interacting loops are disordered (Supplementary Fig. S14e). Compared with the E5-ssDNA-AMPPNP structure, the gap between the SF3 helicase domains of monomers F and A is wider; in addition, the helicase domain of monomer E is completely disordered in the E5_∆N-ssDNA-AMPPNP structure. These observations indicated that AMPPNP binding can induce domain rearrangement of the MPXV E5 protein.

### The C-Tail is dispensable for DNA unwinding by MPXV E5

Although not observed in the MPXV E5-AMPPNP structure, the C-Tail domains (aa 694-785) of monomers B, C and D are ordered in the E5-ssDNA-AMPPNP structure (Fig. 2a). The C-Tail is mainly composed of four α-helices and four β-strands, which arrange into two anti-parallel β-sheets (Supplementary Fig. S16a). In the structure, all C-Tails tilt toward the SF3 helicase domains of the adjacent monomers, but only the C-Tail of monomer C forms one H-bond interaction with the SF3 helicase domain of monomer B (Supplementary Fig. S16b, c). In the E5_∆N-ssDNA-AMPPNP structure (Fig. 4c), only one C-Tail (of monomer A) was observed. In both structures, the C-Tail does not form much interdomain interaction and is not involved in direct DNA binding.

Like MPXV E5, the C-Tail domain is also present in many other SF3 family members, such as *SaPI5*PriRep1^36^, *Cyanophage* S-2L PrimPol, *Sulfolobus islandicus* pRN1, ASFV C962R, and NrS-1 PrimPol. As demonstrated by mutagenesis and/or structural studies, the C-Tail is important for domain arrangement and DNA unwinding by ASFV C962R^33^ and NrS-1 PrimPol^35^. The structural observations promote us to ask whether the C-Tail is also required for DNA unwinding by MPXV E5. To this end, we first performed a structural comparison (Supplementary Fig. S16a). The C-Tail of MPXV E5 and ASFV C962R^33^ are all α/β folds in nature, but the arrangement and orientations of the secondary structural elements are quite different in the two structures. Unlike MPXV E5, the C-Tail of NrS-1 PrimPol is mainly composed of α-helices^35^. We then constructed and purified one MPXV protein with both N-terminal domains and C-Tail truncated (E5_∆NC, aa 323–694). As depicted in Fig. 4e, E5_∆NC can efficiently unwind the forked DNA. At lower concentrations (25 and 50 nM), the DNA unwinding activity of E5_∆NC is even stronger than that of E5_∆N. These observations indicated that the C-Tail is not required; instead, it might have a minor inhibitory effect on DNA unwinding by MPXV E5.

### Verification of functionally important residues of MPXV E5

**Fig. 5:**
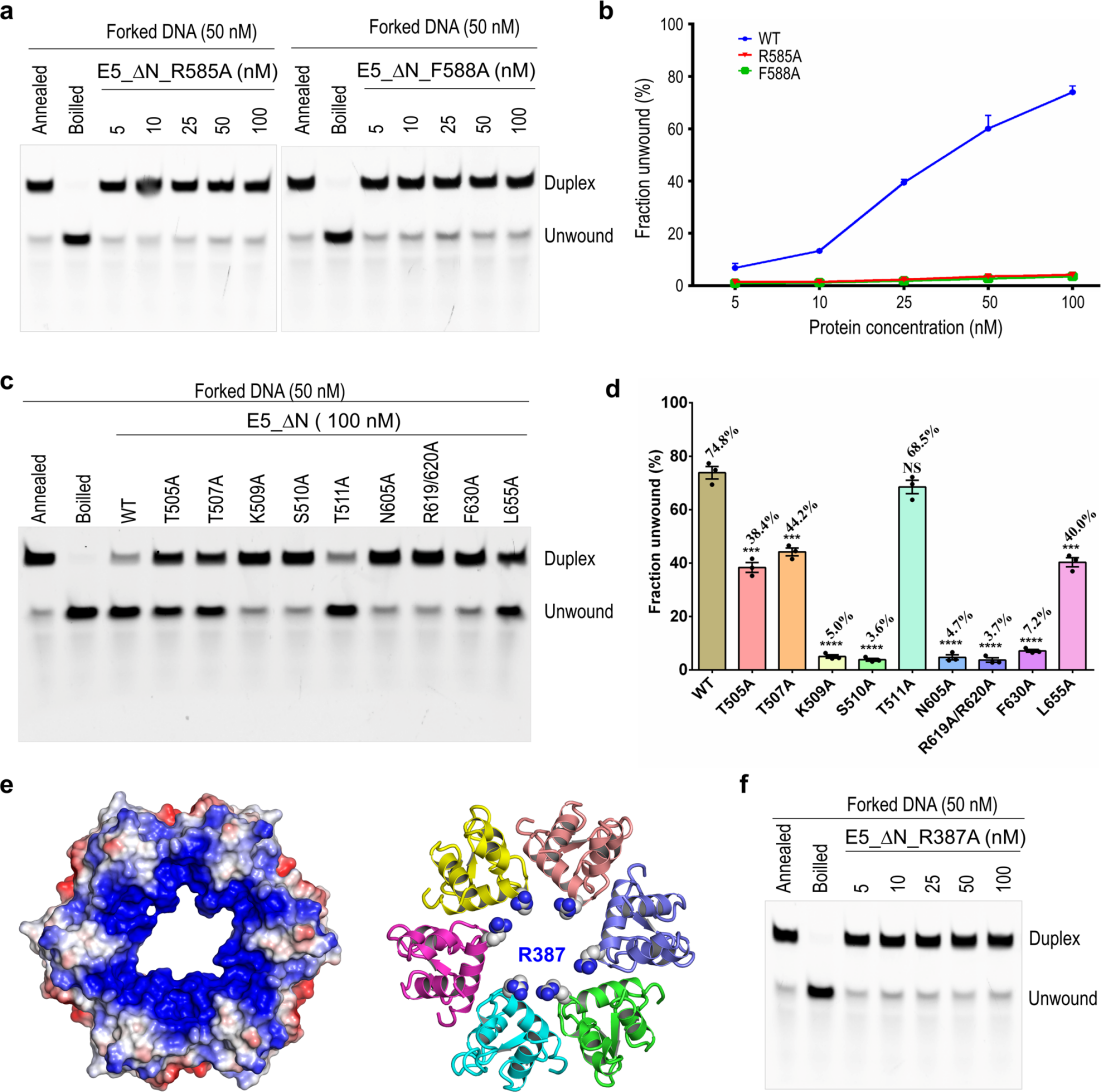
Impacts of MPXV E5 mutation on DNA unwinding. **a**, **b** In vitro DNA unwinding assays catalyzed by E5_∆N mutants with DNA-binding residues mutated. Symbols represent mean ± SEM (*n* = 3). **c**, **d** In vitro DNA unwinding assays catalyzed by E5_∆N mutants with ATP-binding residues mutated. Symbols represent each biological replicate; center values and error bars represent means ± SEM (*n* = 3). ***P < 0.001, ****P < 0.0001, NS means no significance (two-tailed); statistical significance determined by unpaired *t*-test. **e** Surface and cartoon presentation of the D5N domain of MXPV E5. The positive and negative charged residues are colored in blue and red, respectively. **f** In vitro DNA unwinding assays catalyzed by E5_∆N R387A mutant.

Based on the structural observations (Fig. 2c), we constructed two E5_∆N mutants with the DNA-interacting residues Arg585 and Phe588 substituted by Ala, respectively. As depicted in Fig. 5a, b, the DNA unwinding activities of the R585A and F588A mutants are significantly weaker than that of wild-type (WT) E5_∆N. At a concentration of 100 nM, only less than 3% of forked DNA can be unwound by the two mutants, confirming that both Arg585 and Phe588 are important for the DNA unwinding activity of MPXV E5.

The ATPase activity is critical for DNA unwinding by many SF3 family helicases^33,35^. Although the helicase activity has not been demonstrated, a previous study confirmed VACV D5 possesses ATPase activity^22,30^. Based on previous mutagenesis study on VACV D5 and our structural observations, we designed eight E5_∆N mutants with single ATP-binding residue mutated, including T505A, T507A, K509A, S510A, T511A, N605A, Phe630, and Leu655. A double point mutant was also constructed, in which both Arg619 and Arg620 are substituted by Ala (for mutant R619A/R620A). As shown by the in vitro assays (Fig. 5c, d; Supplementary Fig. S17), Thr511 mutation has no obvious impact on DNA unwinding by the E5_∆N protein. However, the unwinding activities of the T505A, T507A and L655A mutants are reduced by 40%–60%, when compared with the WT E5_∆N protein. More dramatic reductions were observed for the K509A, S510A, N605A, R619A/R620A, and F630A mutants, indicating that all these residues play critical roles in DNA unwinding by MPXV E5.

The D5N domain does not form direct interaction with DNA in either of the two DNA-bound MPXV E5 structures. However, careful analysis showed that D5N is positive in charge at the upper edge, due to the presence of Arg387 residues (Fig. 5e). As observed in many reported protein–DNA complex structures, Arg can form electrostatic interaction with the phosphate backbone of DNA^37^. To investigate whether Arg387 plays a certain role in DNA binding and unwinding by MPXV E5, we constructed one E5_∆N R387A mutant. As depicted in Fig. 5f, Arg387 substitution by Ala significantly reduced the DNA unwinding activity of E5_∆N.

The above structural and in vitro assay results confirmed that the SF3 helicase domain of MPXV E5 is functional, but it is inhibited by the N-terminal domains, especially the AEP domain next to the DNA entrance. Structural analysis suggested that auto-inhibition of MPXV E5 is likely mediated by electrostatic interactions, via the negatively charged residues Glu78, Glu79 and Asp81 of the AEP domain and Arg387 of the D5N domains (Supplementary Fig. S18). Because Arg387 mutation will abolish the DNA unwinding activity, we then constructed one MPXV E5 triple mutant with Glu78, Glu79 and Asp81 substituted by Ala. Different from WT MPXV E5, which can be readily expressed and purified in fusion with Mcor-tag, the triple mutants fused with either Mcor-, Sumo- or MBP-tags are expressed in inclusion body and are not suitable for in vitro DNA unwinding assay.

### AEP domain of MPXV E5 possesses high catalytic activity

**Fig. 6:**
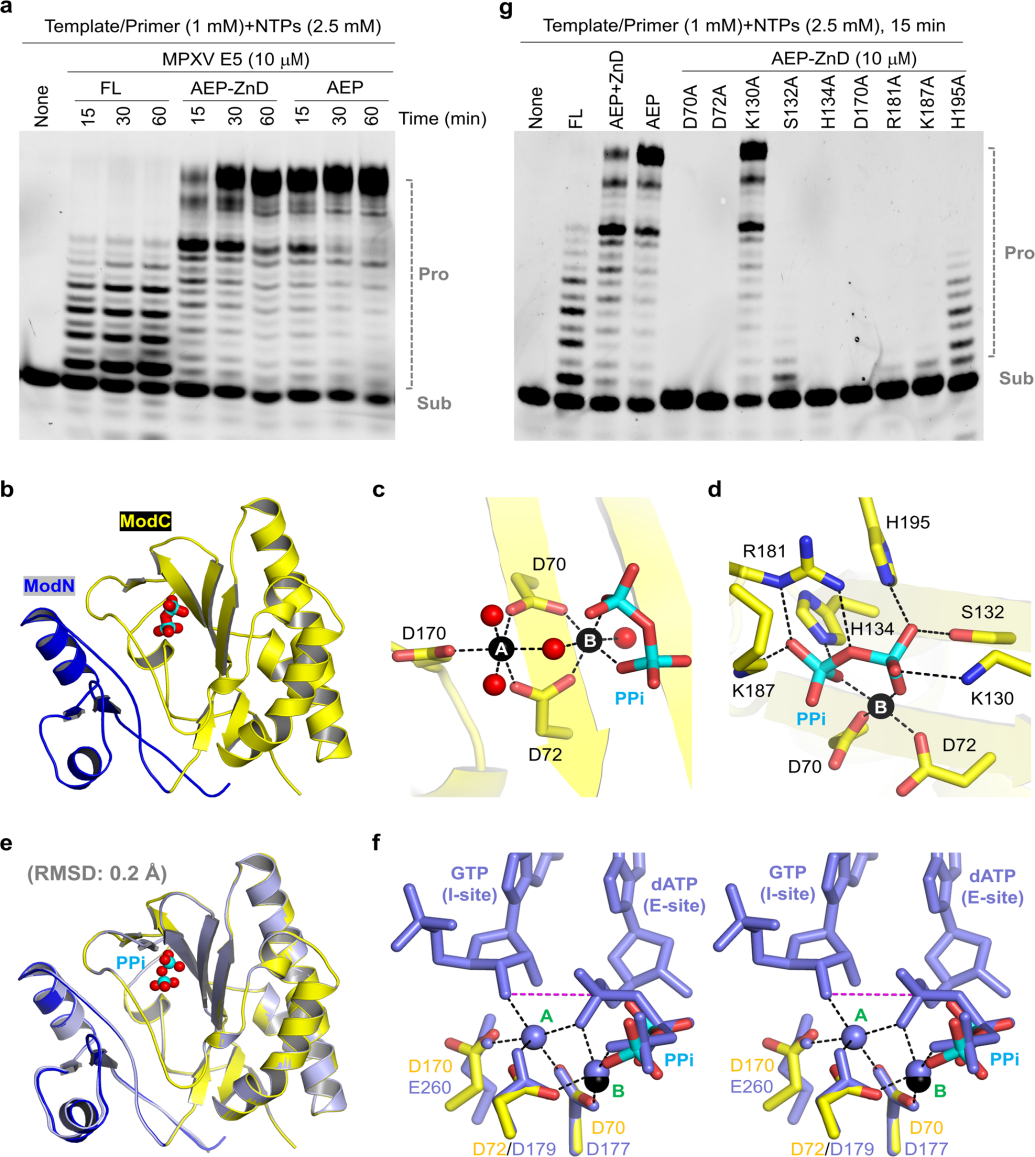
Characterization of the AEP domain of MPXV E5. **a** In vitro primer extension assays catalyzed by the full-length and truncated MPXV E5 proteins. **b** Overall folding of MPXV E5 AEP domain. **c**, **d** Mg^2+^ coordination and PPi recognition in the MPXV E5 AEP domain structure. **e** Superposition of the AEP domains of MPXV E5 and VACV D5. **f** Catalytic site comparison of MPXV E5 AEP domain with the *Ms*CAPP structure. **g** In vitro primer extension assays catalyzed by WT MPXV E5 and mutant proteins. MPXV E5 AEP domain and the bound Mg^2+^ are colored in blue-and-yellow and black in **b**–**f**.

MPXV E5 contains one AEP domain at its N-terminus (Fig. 1a). As demonstrated by the homologous protein D5 in VACV, the AEP domain can catalyze the de novo RNA primer synthesis and is required for the replication of the viral DNA^23^. Two AEP domains were observed in the apo-form MPXV E5 structure, but neither of them binds with nucleic acids (Fig. 1d). To investigate whether MPXV E5 AEP domain is functional, we constructed two truncated proteins (AEP, aa 1–230; AEP-ZnD, aa 1–322) and performed in vitro catalytic assays (Fig. 6a; Supplementary Table S1). Compared to the full-length protein, the catalytic activities of AEP and AEP-ZnD are much higher. With a reaction time of 60 min, no full-length RNA product was observed for the full-length protein. Detectable amount of the full-length product was produced by AEP-ZnD after 15 min reaction time. Under the same conditions and reaction time, AEP produced more than 80% of the full-length products. These observations indicated that the AEP domain of MPXV E5 is highly active in primer synthesis, whereas the ZnD domain is not required for such activity. In the future, it is worth investigating whether the ZnD domain also plays certain biological roles, such as balancing the primase and helicase activities of MPXV E5 in vivo.

To further verify the catalytic activity of MPXV E5, we performed crystallization studies for AEP and AEP-ZnD in the presence of DNA templates and NTPs (Supplementary Table S1). No crystal grew for AEP-ZnD, but one high-resolution AEP structure (Fig. 6b) was determined (1.65 Å, Supplementary Table S3). Supported by the low RMSD value (0.6 Å), the overall foldings of the AEP domains are very similar in the crystal and cryo-EM structures (Supplementary Fig. S19a). No DNA template or RNA product was observed, but clear electron densities confirmed that two Mg^2+^ ions (common factor of DNA and RNA polymerase) and one pyrophosphate group (PPi) were captured in the AEP structure (Supplementary Fig. S19b, c). PPi is the side product of DNA or RNA synthesis^38^. Since no PPi was included in the crystallization sample, the observed PPi should be produced by the AEP-catalyzed de novo synthesis reaction.

Both Mg^2+^ ions are six-coordinated (Fig. 6c). In addition to the side chains of Asp70, Asp72 and Asp170, the A-site Mg^2+^ ion also coordinates with three water molecules. The B-site Mg^2+^ ion coordinates with the side chains of Asp70 and Asp72, two water molecules, and two oxygen atoms of PPi. Besides coordination with Mg^2+^ ion, the conformation of PPi was further stabilized by H-bond interactions with the side chains of Lys130, Ser132, His134, Arg181, Lys187, and His197 (Fig. 6d).

In addition to the AEP domain of MPXV E5, we also performed crystallization study for the AEP domain of VACV D5 and solved one structure at 1.4-Å resolution (Supplementary Table S3). Similar to E5 AEP, one PPi was captured in the D5 AEP structure (Supplementary Fig. S19d). As revealed by structural superposition, the overall folding of E5 and D5 AEP domains are virtually identical (Fig. 6e); the RMSD value between the two domains is only 0.2 Å. These observations indicated that the folding and catalytic activity of the AEP domains are highly conserved in MPXV E5, VACV D5, and the helicases from other orthopoxvirus viruses.

### The catalytic mechanism of MPXV E5 is conserved

The nucleic acid and/or NTP-bound structures have been reported for many AEP domain-containing proteins, such as *Hs*PrimPol (PDB_ID: 5l2x)^39^, *Ms*CAPP (PDB_ID: 7P9J)^40^, and ASFV C962R (PDB_ID: 8IQD)^33^. To gain more insights into the catalytic mechanism of MPXV E5, we performed sequence alignment. As depicted in Supplementary Fig. S20a, the Mg^2+^-coordinating residues (Asp70, Asp72 and Asp170) and two PPi-interacting residues (His134 and Lys187A) are highly conserved, but Lys130, Ser132, Arg181, and His195 are less conserved. Superposition showed that the overall foldings of the AEP domains are similar in the MPXV E5, *Hs*PrimPol and *Ms*CAPP structures (Supplementary Fig. S20b, c).

Besides divalent cations, two nucleotides were also captured at the active site of the *Ms*CAPP structure, which represents the initiation state of the de novo RNA synthesis reaction^40^. The conformations and orientations of cations and the cation-coordinating residues are virtually identical in MPXV E5 AEP and the *Ms*CAPP structure (Fig. 6f). PPi in the MPXV E5 structure well mimics the β- and γ-phosphate groups of the incoming site (E-site) dATP in the *Ms*CAPP structure. The similar structural arrangements suggested that the AEP domain of MPXV E5 follows the conserved two-cation-assisted, in-line attacking mechanism in catalysis^41^, in which A-site cation activates the initiating site (I-site) NTP (or dNTP) by removing the proton from the 3′-OH group and B-site cation facilitates the reaction by increasing the electrophilicity of the phosphorus center. The catalytic mechanism of MPXV E5 can be further supported by the mutagenesis and in vitro catalytic assay results (Fig. 6g). In contrast to the WT protein, no catalytic activity was observed for any of the three mutants (D70A, D72A, and D170A) with the catalytic residues mutated. Substitution of Lys130 by Ala (for K130A) has no impact on the catalytic reaction. However, mutation of His134, Lys187 or other PPi-interacting residues all lowered the catalytic activity, indicating that they are critical for the function of MPXV E5.

## Discussion

In conclusion, we solved three cryo-EM structures of MPXV helicase protein E5 in this study. The MPXV E5-AMPPNP structure shows that the full-length MPXV E5 adopts an asymmetric, auto-inhibited conformation with its AEP domain blocking DNA from entering the central channel in correct orientation (Fig. 1b). The ssDNA bound in the E5-ssDNA-AMPPNP complex most likely enters from the bottom of the channel and cannot be unwound by MPXV E5 (Fig. 2a). Truncation of the N-terminus allows the MPXV E5 protein to show in vitro DNA unwinding activity for the first time (Fig. 4b). In consistent with previous studies on the conditional lethal mutants of VACV D5^42^, MPXV E5 preferentially unwinds forked DNA, suggesting that the helicase proteins of poxviruses function at the replication fork site in vivo.

In addition to DNA template, a short de novo-synthesized RNA primer is also required for the replication of the viral genome. In vitro studies showed that the AEP domain of MPXV E5 is highly activity and can efficiently extend the primer from the 3′-end (Fig. 6a). The de novo primer synthesis activity can be implicated by the PPi molecule observed in the crystal structure of the AEP domain of MPXV E5 (Fig. 6b). Identical structures (Fig. 6e) indicated that the overall folding and catalytic activity of the AEP domains are conserved in MPXV E5, VACV D5, and, most likely, in all other poxviruses members.

Sequence and structural similarities suggested that the primer synthesis, DNA binding and/or unwinding mechanisms are conserved in many other homologous proteins, such as ASFV C962R, *Ms*CAPP, and NrS-1 (Supplementary Figs. S11 and S20). In vitro primer synthesis and DNA unwinding activities have been previously demonstrated for the full-length ASFV C962R^33^ and NrS-1^35^; and these two activities are correlated with each other. The fusion of primer synthesis and helicase activity in one single protein may facilitate the replication process of the viruses. The auto-inhibited conformation sets MPXV E5 apart from these homologous proteins.

Of note, while our manuscript was under review, one similar work on MPXV helicase was reported by Li and co-workers^43^. In their study, they reported several cryo-EM structures. Three AEP domains were observed in one of the structures (PDB_ID: 8HWA). Although one AEP domain was disordered in our MPXV E5-AMPPNP structure, the orientations of the other two AEP domains are virtually identical in our structure and the 8HWA structure (Supplementary Fig. S21). We failed to obtain any mutant to rescue the in vitro DNA unwinding activity of the full-length protein, but they purified and confirmed the DNA unwinding activity of one mutant with a triple-mutation in the AEP domain. Together with their and our structural observations, these in vitro assay results further confirmed that the DNA unwinding activity is auto-inhibited by the N-terminal AEP domains. A previous study showed that the homologous proteins of E5 and A22 can interact with each other in VACV^19^. However, A22 alone is unstable; it requires the assistance of other factors, such as E4 and F8 to be stabilized^44^. In the future, it is worth investigating whether A22 or other protein factors are required for the activation of MPXV E5 in vivo.

In addition to the 8HWA structure, Li and co-workers also reported one ssDNA and ATP-γ-S bound structure (PDB_ID: 8HWG; resolution: 3.0 Å)^43^. Like AMPPNP, ATP-γ-S is also a non-hydrolyzable ATP analog. AMPPNP and ATP- γ-S can not support the DNA unwinding activities, but they can well mimic ATP in interaction and have been widely used in the structural and mechanistic studies of ATP-dependent helicases^33^. Except for the SF3 domains of the monomers E and F, the orientations of other domains are very similar in our E5-ssDNA-AMPPNP structure and the 8HWG structure (Supplementary Fig. S22a). In both structures, the monomers A–D bind the ligand and mimic the ATP-bound state (Supplementary Fig. S22b). Due to the disordering of the γ-phosphate groups of the ligands, the monomers E and F represent an ADP-bound state (Supplementary Fig. S22c). In the ADP-bound state, the two adjacent monomers can undergo obvious conformational changes.

**Fig. 7:**
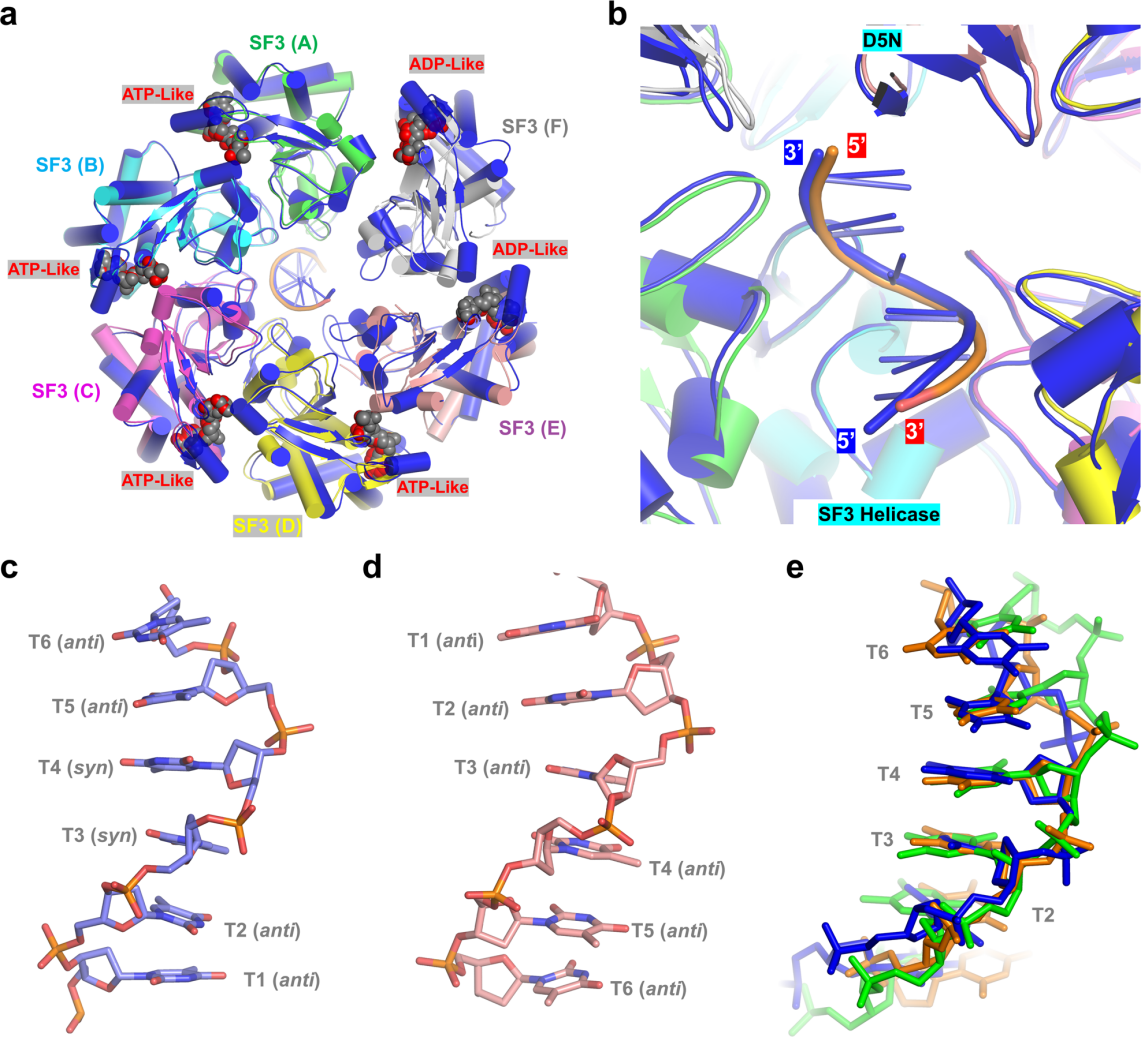
Clarification of the polarity of the DNA bound by MPXV E5. **a**, **b** Superposition of our E5-ssDNA-AMPPNP complex and the reported 8HWG structure, in which both helicase and the bound DNA are colored in blue. **c**, **d** Conformation of the DNAs modeled in the 8HWG structure and our E5-ssDNA-AMPPNP complex, respectively. **e** Superposition of the B-form DNA with these in 8HWG and our E5-ssDNA-AMPPNP structures, which are colored in green, blue, and orange, respectively.

MPXV E5 belongs to the SF3 helicase superfamily, which normally forms hexamer and unwinds DNA with 3′- to 5′ polarity^32^. In the 8HWG structure, the helicase forms hexamer and binds DNA in its central channel (Fig. 7a); however, the orientation of the bound ssDNA is different from that in our structures. Instead of the SF3 helicase domain, the 3′ end of ssDNA points toward the D5N domain in the 8HWG structure (Fig. 7b). To clarify the polarity of the DNA bound by MPXV E5, we performed careful structural analysis. Unlike regular A- and B-form DNAs, the two central nucleotides of the DNA adopt *syn* conformation in the 8HWG structure (Fig. 7c); superposition also showed that the phosphate backbones of the DNA are severely twisted (Fig. 7e), leading to many unfavorable close contacts (Supplementary Fig. S23). In contrast, all nucleotides adopt regular anticonformation and do not form any close contacts in our structures; the central nucleotides can well match with B-form DNA (Fig. 7d, e). Compared to the 8HWG structure, the electron density maps of the DNA are better defined in our E5_∆N-ssDNA-AMPPNP structure. Although the in vitro helicase assay results (Fig. 4b) are not sufficient to directly fix the unwinding direction of MPXV E5, the electron density maps (Supplementary Fig. S14b), geometry analysis (Fig. 7c–e), and similarity with other SF3 family members (Fig. 3) all suggested that MPXV E5 unwinds DNA from the 3′-end and the orientation of the DNA in our structures is more favorable.

VRAV, MPXV, CPXV, and VACV can all infect humans^45^. Due to its relatively low pathogenicity, VACV has been used for many years to vaccinate people to against smallpox. However, more and more pieces of evidence show that VACV infection can cause diseases in some farm animals and humans^46,47^. Therefore, VACV vaccination has been stopped after the eradication of the VRAV. The recent emergency of MPXV emphasizes the urgent need for developing safe and efficient drugs for poxviruse treatment.

As a member of poxviruses, the genome of MPXV solely replicates in the cytoplasm of the host cell^48^. Different from many other DNA viruses, poxviruse does not depend on nuclear enzymes from the host; most, if not all, the proteins necessary for genome replication are encoded by their own genome. Like polymerase, processivity factor, uracil DNA glycosidase, phosphoprotein, and the single-stranded DNA-binding protein, the helicase protein is also encoded by the viral genome and critical for the replication of poxviruses. In addition, the helicase also plays a critical role in the uncoating process of the viral genome. A previous study has shown that silencing helicase D5 by an anti-D5 siRNA can significantly reduce virus production in a VACV-infected mouse model^24^. These observations clearly indicated that the helicase is an ideal target for drug development.

Many small molecule inhibitors have been designed to target the proteins important for the life cycle of MPXV, such as the DNA ploymerase, Topisomerase 1, and Cysteine proteinases^49,50^. However, these molecules mainly target the catalytic site of the protein, which is largely conserved in the homologous proteins in the host. Since the auto-inhibited conformation of MPXV E5 is unique, fixing the conformation of MPXV E5 at the inhibited state by antibodies or small molecules will be a good strategy to treat MPXV. Owing to the high sequence and structural similarity of the helicases, such antibodies and small molecules can also be used to treat other poxviruses. Our studies not only advance our understanding on the biological function of MPXV E5, but also provide an ideal structural basis for the development of drugs against poxviruses.

## Materials and methods

### Plasmid construction, protein expression, and purification

The gene containing the codon-optimized cDNA sequences of MPXV E5 (aa 1–785, UniProt ID: A0A7H0DN89) and VACV D5 (aa 1–322) (Supplementary Table S4) were purchased from Beijing Tsingke Biotech Co., Ltd., China. The target fragment was recombined into the pET-His6-Mocr and pET-28a-SUMO vector, respectively. The recombinant His6-Mocr-MPXV E5 coding vector was utilized as the template during the plasmid constructions of truncated MPXV E5 proteins. All MPXV E5 mutant proteins were constructed using a Homologous Recombination kit (ClonExpress® Ultra One Step Cloning Kit, Vazyme), the detailed sequences of the primers were listed in Supplementary Table S5. All plasmids were transformed into *E. coli* BL21(DE3) competent cells for protein expression. Sequences of all plasmids were confirmed by DNA sequencing.

All MPXV E5 and VACV D5 proteins were expressed and purified using the same procedures. Briefly, the frozen recombinant strains were revived in Lysogeny broth (LB) medium supplemented with 50 μg/mL kanamycin at 37 °C overnight. Every 15 mL revived bacterium suspension was inoculated into 1 L LB medium and cultured at 37 °C. Protein expression was induced at OD_600_ ≈ 0.6 by adding isopropyl β-D-1-thiogalacto-pyranoside at a final concentration of 0.2 mM. The induced cultures were grown at 18 °C for an additional 20 h. The cells were collected via centrifugation, resuspended in Buffer A (20 mM Tris pH 8.0, 250 mM NaCl, 25 mM imidazole), and lysed under high pressure. The supernatant was loaded onto a HisTrap™ HP column and washed with Buffer B (20 mM Tris pH 8.0, 2 M NaCl). The target protein was eluted via ÄKTA pure (Cytiva) system using Buffer C (20 mM Tris pH 8.0, 250 mM NaCl, 500 mM imidazole). The proteins were treated with ULP or TEV protease for 30 min or 6 h. The sample was re-loaded onto the HiTrap™ SP HP or HiTrap™ Heparin HP column using Buffer D (20 mM Tris pH 8.0, 100 mM NaCl). The target protein was eluted using Buffer E (20 mM Tris pH 8.0, 1 M NaCl), concentrated, and loaded onto a Superose 200 Increase 10/300 GL column (Cytiva) equilibrated with Gel Filtration Buffer F (20 mM Tris pH 8.0, 200 mM NaCl). All proteins were analyzed using SDS-PAGE gel, concentrated by centrifugal concentrator (Millipore, Burlington, MA, USA), and stored at –80 °C until use.

### Negative stain EM

The full-length MPXV E5 protein collected from the peak of the Superose 200 Increase 10/300 GL column was diluted to 40 μg/mL using Buffer F. 5 µL of proteins were applied to the glow-discharged 200 mesh carbon-coated copper grids (Beijing Zhongjingkeyi Technology). The samples were stained using 0.75% uranyl formate and air-dried. Data were collected on a Talos L 120 C transmission electron microscope equipped with a 4 K × 4 K CETA CCD camera. Images were recorded at a nominal magnification of 73,000×, corresponding to a pixel size of 1.95 Å.

### Cryo-EM sample preparation, data collection, and image processing

MPXV E5/DNA or E5_∆N/DNA samples used for cryo-EM analysis were dissolved in Buffer F. The protein was mixed with 35 bp forked DNA (Supplementary Table S1) and incubated at room temperature for 1 h. The mixture was then injected to the Superose 200 Increase 10/300 GL column, the peak was collected and concentrated to ~10 mg/mL. AMPPNP was added to a final concentration of 1 mM. For cryo-EM grid preparation, 3 μL MPXV E5/DNA or E5_∆N/DNA samples (~10 mg/mL) were applied onto glow-discharged holey carbon grids (Quantifoil Cu R1.2/1.3, 300 mesh), blotted with a Vitrobot Marker IV (Thermo Fisher Scientific) for 3 s under 100% humidity at 4 °C, and subjected to plunge freezing into liquid ethane. All cryo-EM data were collected using the FEI Titan Krios microscope at 300 kV equipped with a Gatan K3 Summit direct electron detector (super-resolution mode, at a nominal magnification of 81,000) and a GIF-quantum energy filter. Defocus values were set from –1.0 to –2.0 μm. Each stack of 32 frames was exposed for 1.56 s, with a total electron dose of 50 e^–^/Å2. EPU (Thermio Fisher Scientific) was used for fully automated data collection.

### EM data processing, model building, and validation

All micrograph stacks were motion corrected with MotionCor2^51^ with a binning factor of 2, resulting in a pixel size of 1.0773 Å or 1.081 Å, indicated on the EM data processing flowchart respectively. Cryo-EM image processing was performed using cryoSPARC^52^. For 3D processing of the MPXV E5/DNA data, a total of 13,074,438 particles were automatically picked from 5239 micrographs. Particles were extracted with a pixel size of 4.3092 Å and subjected to several rounds of reference-free 2D classification. 1,185,068 particles were kept after the exclusion of obvious ice contamination and junk particles, and subsequently reextracted without binning. Then, ab initio models were generated and subsequently used for heterogeneous 3D refinement. The class of 126,172 particles for apo-form MPXV E5 were then used for further non-uniform refinement and local refinement and were used for further structural analysis; 221,856 particles for MPXV E5/DNA complex were used to generate ab initio models and subsequent heterogeneous 3D refinement, the class of 221,856 particles were then used for further homogenous refinement, non-uniform refinement and local refinement, and was used for further structural analysis.

For 3D processing of the MPXV E5_ΔΝ/DNA data, a total of 11,718,083 particles were automatically picked from 2913 micrographs. Particles were extracted with a pixel size of 4.324 Å and subjected to several rounds of reference-free 2D classification. 2,581,865 particles were kept after the exclusion of obvious ice contamination and junk particles, subsequently reextracted without binning. Then, two rounds of ab initio-heterogeneous 3D refinement were performed. The class with 247,898 particles for MPXV E5_ΔΝ/DNA complex was used for homogenous refinement, nonuniform refinement, and local refinement, and the resulting cryo-EM map was used for further structural analysis.

The model of the MPXV E5 monomer was predicted by AlphaFold2^53^ and docked into EM 3D density maps using the program ChimeraX^54^. The model adjustment was manually done in ChimeraX and COOT^55^. The resulting models were refined against the EM map by PHENIX^56^ in real space with secondary structure and geometry restraints. The final models were validated in the PHENIX software package. The model statistics are summarized in Supplementary Table S2.

### Crystallization, data collection and structural refinement

The isolated AEP and AEP-ZnD domains of MPXV E5 and VACV D5 were purified and utilized in the crystallization screen. For the complex formation, the protein, magnesium ion, NTPs, and Template ssDNA (Supplementary Table S1) were mixed; the final protein, manganese ion, NTPs, and Template ssDNA concentrations are 86 mg/mL, 10, 10, and 5 mM, respectively. The initial crystallization conditions were identified by the sitting-drop vapor-diffusion method using commercial crystal screening kits at 18 °C. The drop contained an equal volume (0.2 μL) of protein sample and reservoir solution and was equilibrated against 50 μL of reservoir solution in a 96-well format. The reservoir solutions are composed of 0.1 M Magnesium formate dihydrate, 15% w/v Polyethylene glycol 3,350.

All crystals were cryoprotected using their mother liquor supplemented with 25% glycerol and snap-frozen in liquid nitrogen. The diffraction data were collected at beamlines BL10U2 at the Shanghai Synchrotron Radiation Facility (SSRF) and beamline BL18U1 of the National Facility for Protein Science Shanghai (NFPS). Data processing was carried out using the xia2_3dii or HKL3000^57^ program. The data collection and processing statistics were summarized in Supplementary Table S3. The AEP structure was solved by molecular replacement (MR) method using the model predicted by AlphaFold2 and the Phaser program^58^ of the CCP4 suite^59^. The resulting models were refined against the diffraction data using the Refmac5 program of the CCP4 suite. The 2F_o_–F_c_ and F_o_–F_c_ electron density maps were regularly calculated and used as guide for the building of water, ions, and PPV in COOT. The final refinement of all structures was performed using the phenix.refine program. The structural refinement statistics were also summarized in Supplementary Table S3.

### DNA unwinding assay

The Top and the Bottom strands of 5′- or 3′-overhangs DNA or forked DNA (Supplementary Table S1) were mixed with a molar ratio of 1:1. The mixtures were heated at 95 °C for 5 min, followed by slow cooling to room temperature. The annealed DNAs (50 nM) were then mixed with full-length or truncated MPXV E5 proteins in the buffer composed of 20 mM Tris pH 8.0, 100 mM NaCl, 10 mM MgCl_2_, 10 mM ATP, and 2 mM DTT. To prevent the re-annealing of the unwound Top strand and Bottom strand, 5 μM of non-labeled Bottom strand DNA was also included in the reaction system. After incubation at 37 °C for 60 min, the reaction was terminated by adding EDTA to a final concentration of 20 mM. The protein was digested by 5 mg/mL Proteinase K at room temperature for 20 min. Samples were then loaded onto 10% TBE polyacrylamide gel for electrophoresis on ice. The gel was visualized using Typhoon FLA 9000. All experiments were performed in triplicate, and the data were presented as means ± SEM by GraphPad Prism 6.

### DNA primer extension assay

The Template strand and the FAM-labeled RNA Primer strand of DNA/RNA (Supplementary Table S1) were mixed with a molar ratio of 1:1 in Buffer composed of 20 mM Tris pH 8.0, 100 mM NaCl. The mixtures were heated at 95 °C for 5 min, followed by slow cooling to room temperature. The annealed DNA/RNA (1 μM) was then mixed with the full-length MPXV E5 protein or the isolated AEP, AEP-ZnD or AEP-ZnD mutants in buffer composed of 20 mM Tris pH 8.0, 100 mM NaCl, 10 mM MgCl_2_, 2.5 mM NTPs and 2 mM DTT. The reaction mixtures were incubated at room temperature. At specific time points, 5 μL aliquots of the reaction were quenched with 20 μL Formamide loading buffer (90% formamide, 20 mM EDTA, 0.05% bromophenol blue, and 0.05% xylene blue) and boiled at 95 °C for 5 min. Samples were loaded onto pre-warmed 16% urea sequencing gels and run for 2 h. The gel was visualized using Typhoon FLA 9000.

### Supplementary information

Supplementary information, Figures and Tables

## Data Availability

Structural factors and coordinates have been deposited in the Protein Data Bank under accession codes 8XIF and 8XIG for the E5 AEP domain structure and the D5 AEP domain structure, respectively. Structural coordinates have been deposited in the Protein Data Bank under accession codes 8XJ6, 8XJ7, and 8XJ8 for the full-length apo-form MPXV E5 structure, MPXV E5/DNA complex and the E5_∆N/DNA complex, respectively. The sequences of the optimized MPXV E5 and VACV D5 genes are listed in Supplementary Table S4. The primers used for mutagenesis are listed in Supplementary Table S5.
