# Supplementary material for: Structural and functional insights into the helicase protein E5 of Mpox virus
Source: Cell Discov. 2024 Jun 25;10:67. doi: 10.1038/s41421-024-00680-1 (PMC11196578; doi:10.1038/s41421-024-00680-1)
Supplement: Supplementary file 1 — Supplementary information, Figures and Tables [file 41421_2024_680_MOESM1_ESM.pdf]

Supplementary Information

for

Structural and functional insights into the helicase protein E5 of Mpox virus

Weizhen Zhang[1]†, Yusong Liu[1,2,3]†, Mengquan Yang[2,3], Jie Yang[1], Zhiwei Shao[1], Yanqing Gao[1], Xinran Jiang[1], Ruixue Cui[4], Yixi Zhang[1], Xin Zhao[1], Qiyuan Shao[1], Chulei Cao[1], Huili Li[1], Linxi Li[1], Hehua Liu[1], Haishan Gao[2,3]*, Jianhua Gan[1,]*

Corresponding author: gaohaishan@westlake.edu.cn; ganjhh@fudan.edu.cn

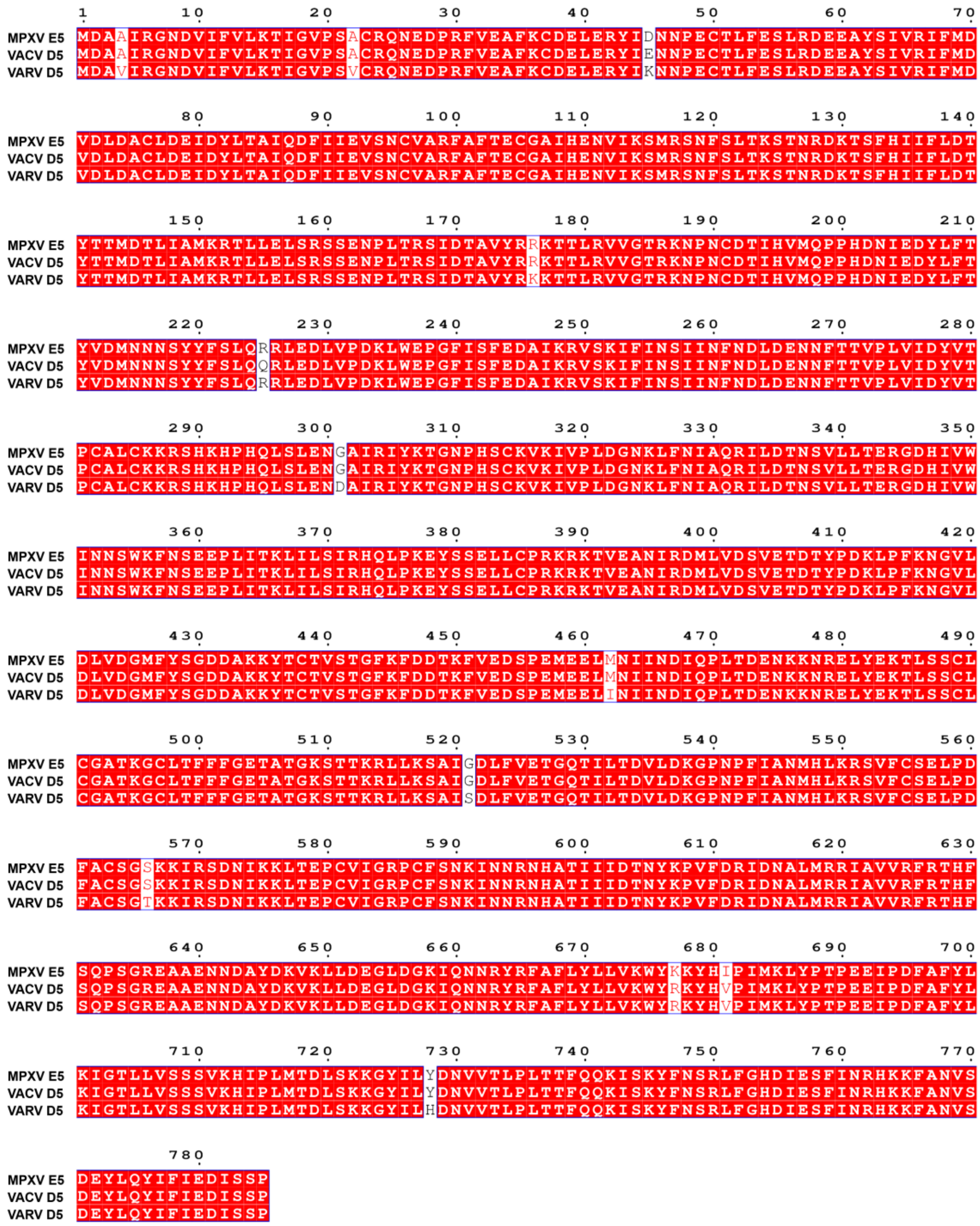

Supplementary Fig. S1 Sequence alignment of the helicase proteins of orthopoxvirus.

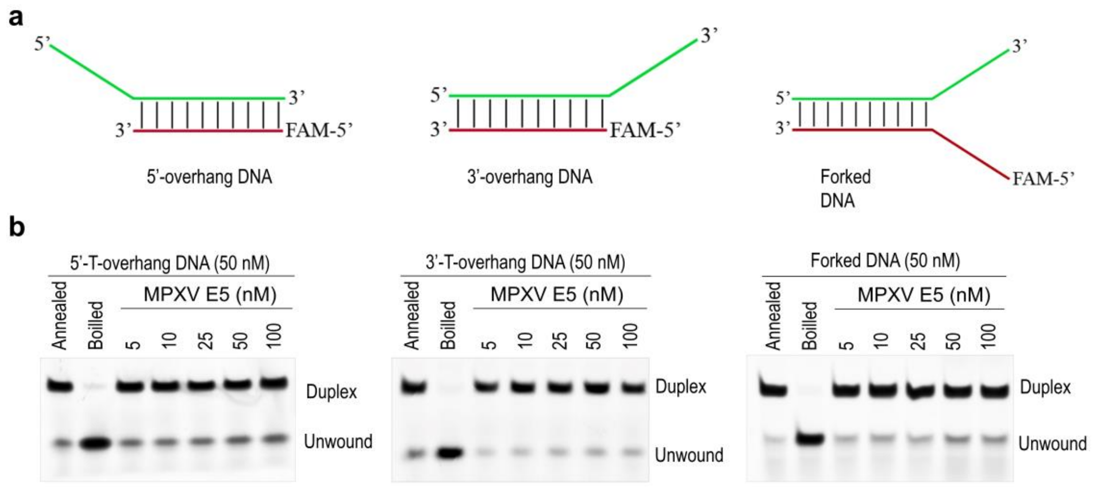

Supplementary Fig. S2 Verification of *in vitro* DNA unwinding activity of MPXV E5. a Schemes of the DNA substrates used in the unwinding assays. **b** *In vitro* DNA unwinding assays catalyzed by full-length MPXV E5 protein.

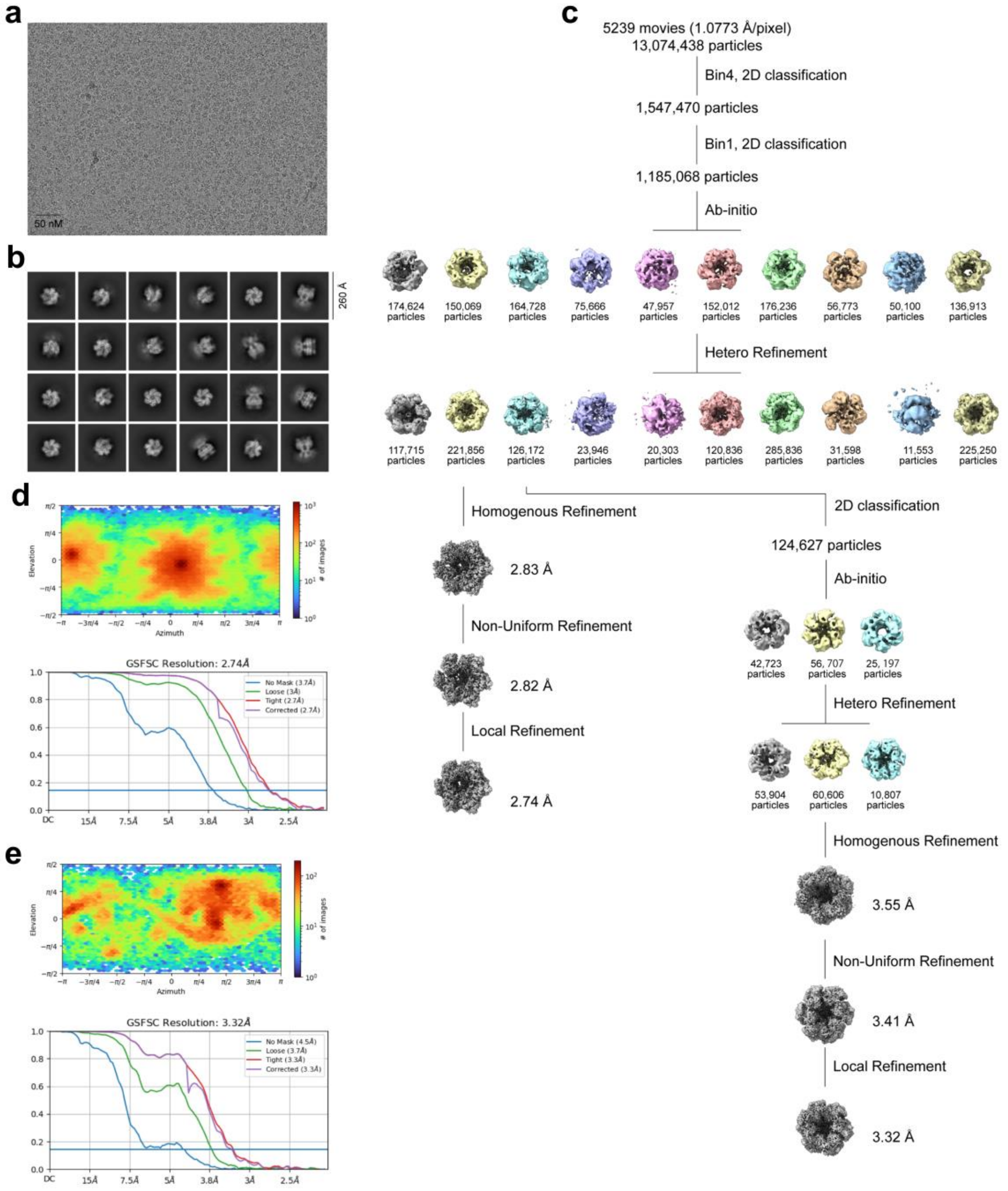

Supplementary Fig. S3 Cryo-EM data processing of the AMPPNP and DNA-bound full-length MPXV E5 structures. a A representative cryo-EM image of the two MPXV E5 structures. **b** Representative 2D-classification images of the two MPXV E5 structures. **c** Workflow of cryo-EM data processing. **d** Particle distribution and Gold-standard Fourier shell correlation (GSFSC) of the final map of the DNA-bound MPXV E5 structure. **e** Particle distribution and Gold-standard Fourier shell correlation (GSFSC) of the final map of the apo-form MPXV E5 structure.

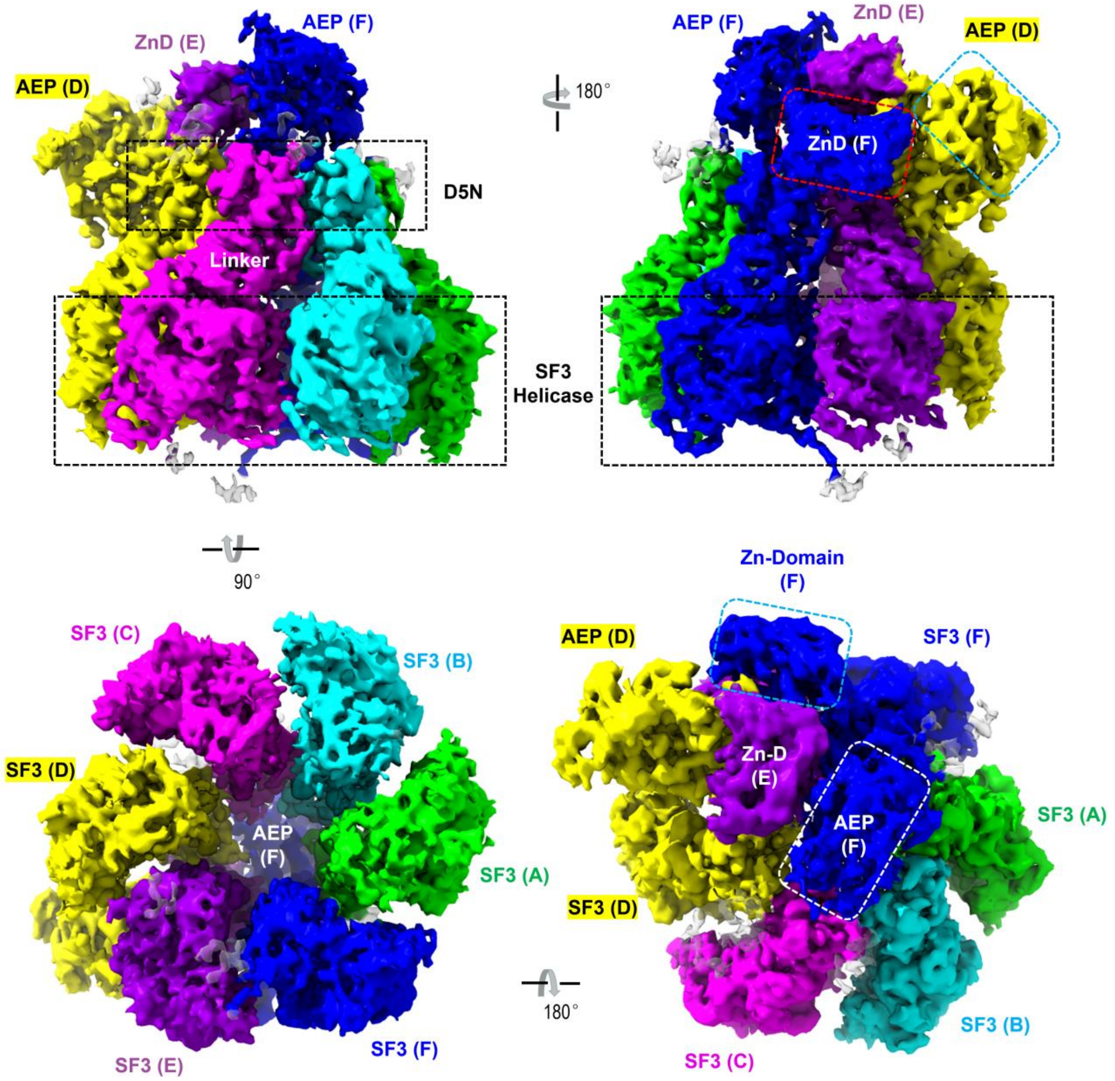

Supplementary Fig. S4 The final electron density map of the MPXV E5-AMPPNP structure. The monomers A to F are colored in green, cyan, magenta, yellow, dark purple, and blue, respectively.

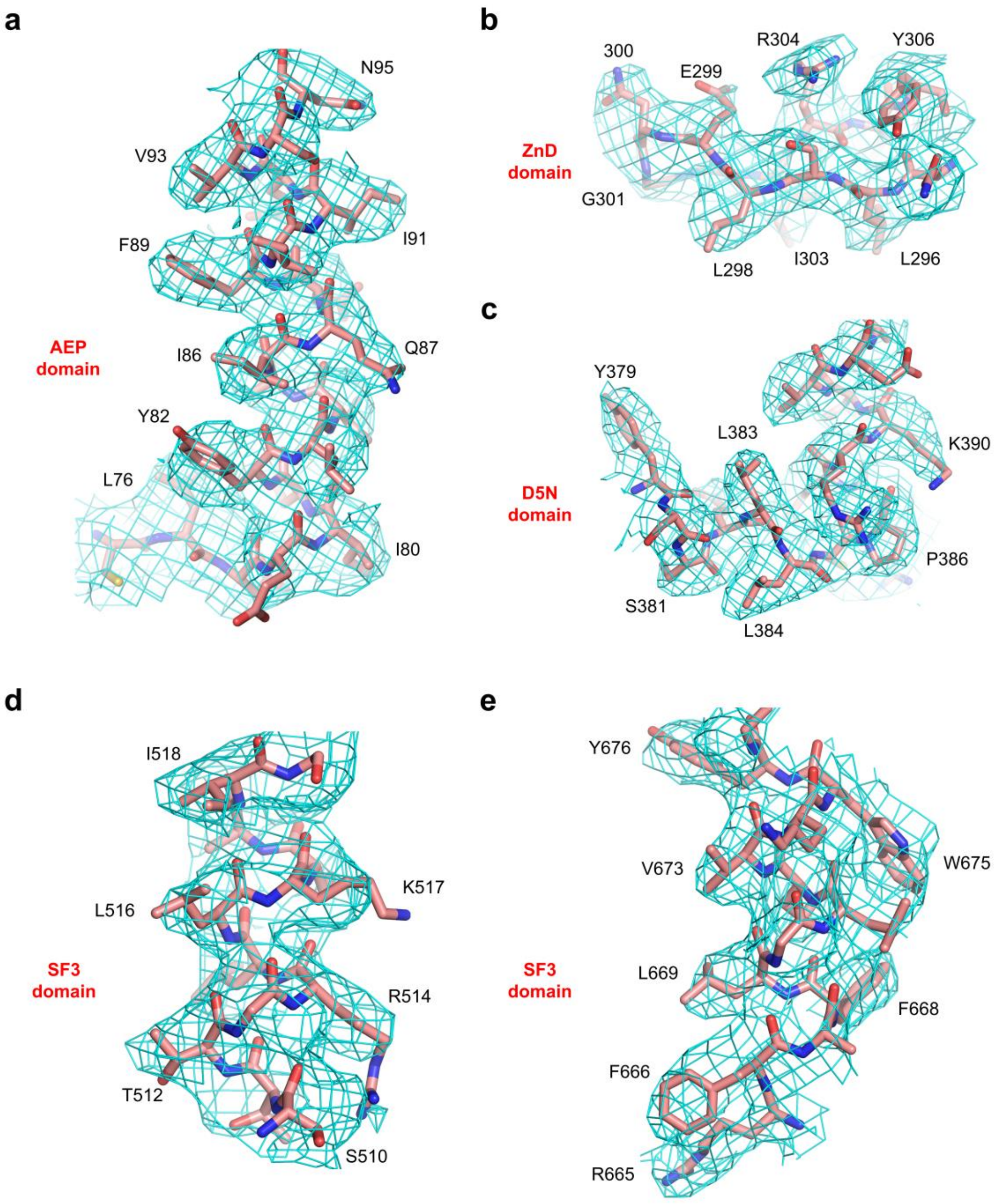

Supplementary Fig. S5 Representative cryo-EM density maps of MPXV E5 protein. a-c The electron density maps for residues from the AEP, ZnD, and D5N domains, respectively. **d-e** The electron density maps for residues from the SF3 domain.

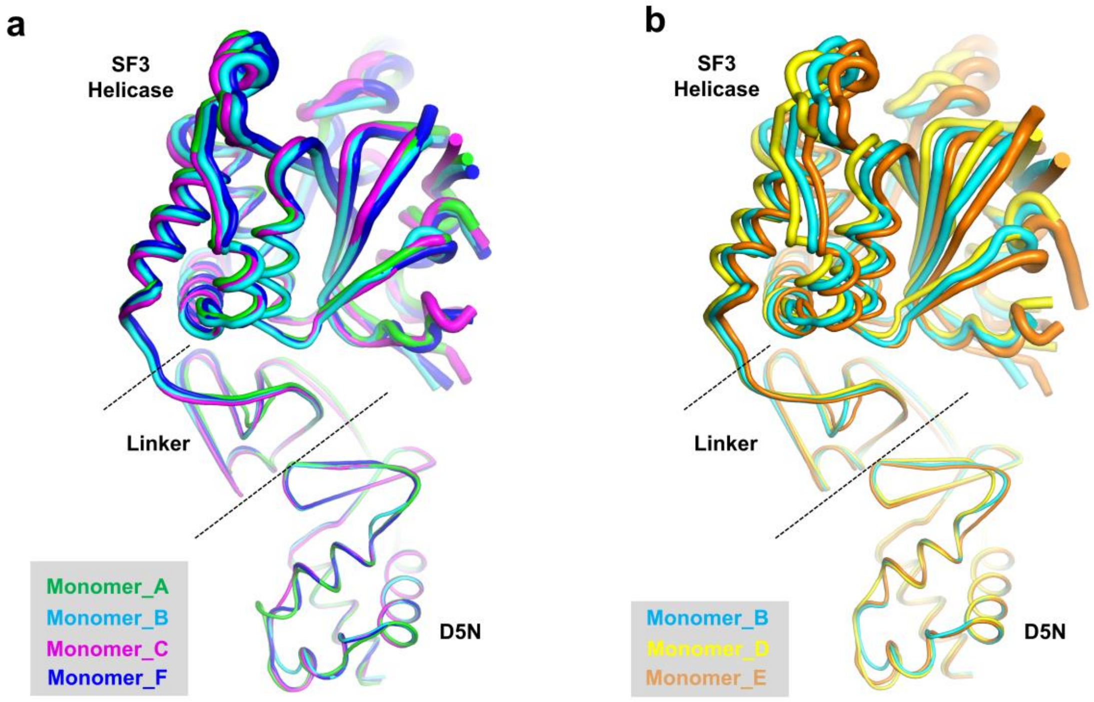

Supplementary Fig. S6 Conformational comparison of MPXV E5 monomers in the E5-AMPPNP structure. a Superposition of MPXV E5 monomers A, B, C, and F. **b** Superposition showing the orientational difference between the monomers D and E and other monomers. For clarity, monomers A, C, and F are not shown.

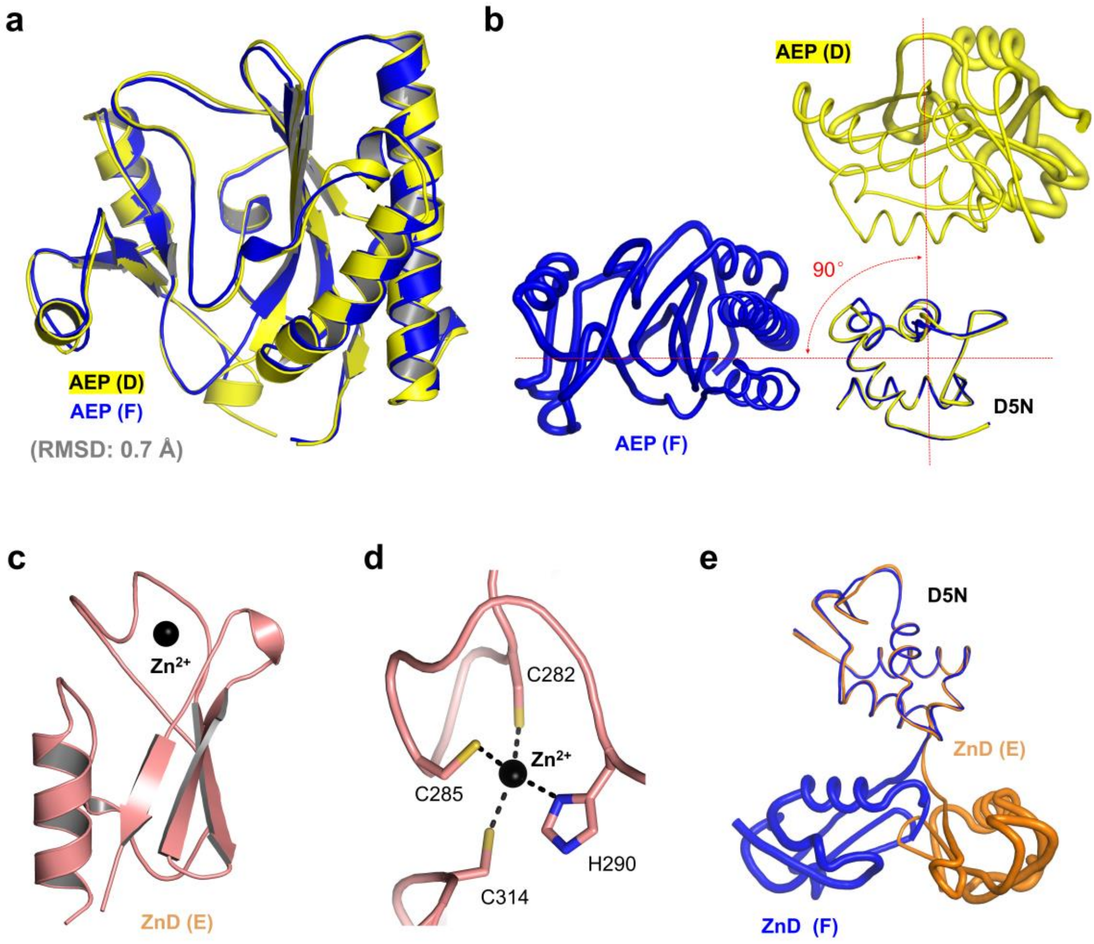

Supplementary Fig. S7 Folding and comparison of individual domains in the MPXV E5-AMPPNP structure. a Superposition of the AEP domains of MPXV E5 monomers D and F. **b** Superposition showing the orientational difference between the two AEP domains. **c** Overall folding of the ZnD domain. **d** The detailed Zn^{2+}-coordination by the ZnD domain. **e** Superposition showing the orientational difference between the two ZnD domains.

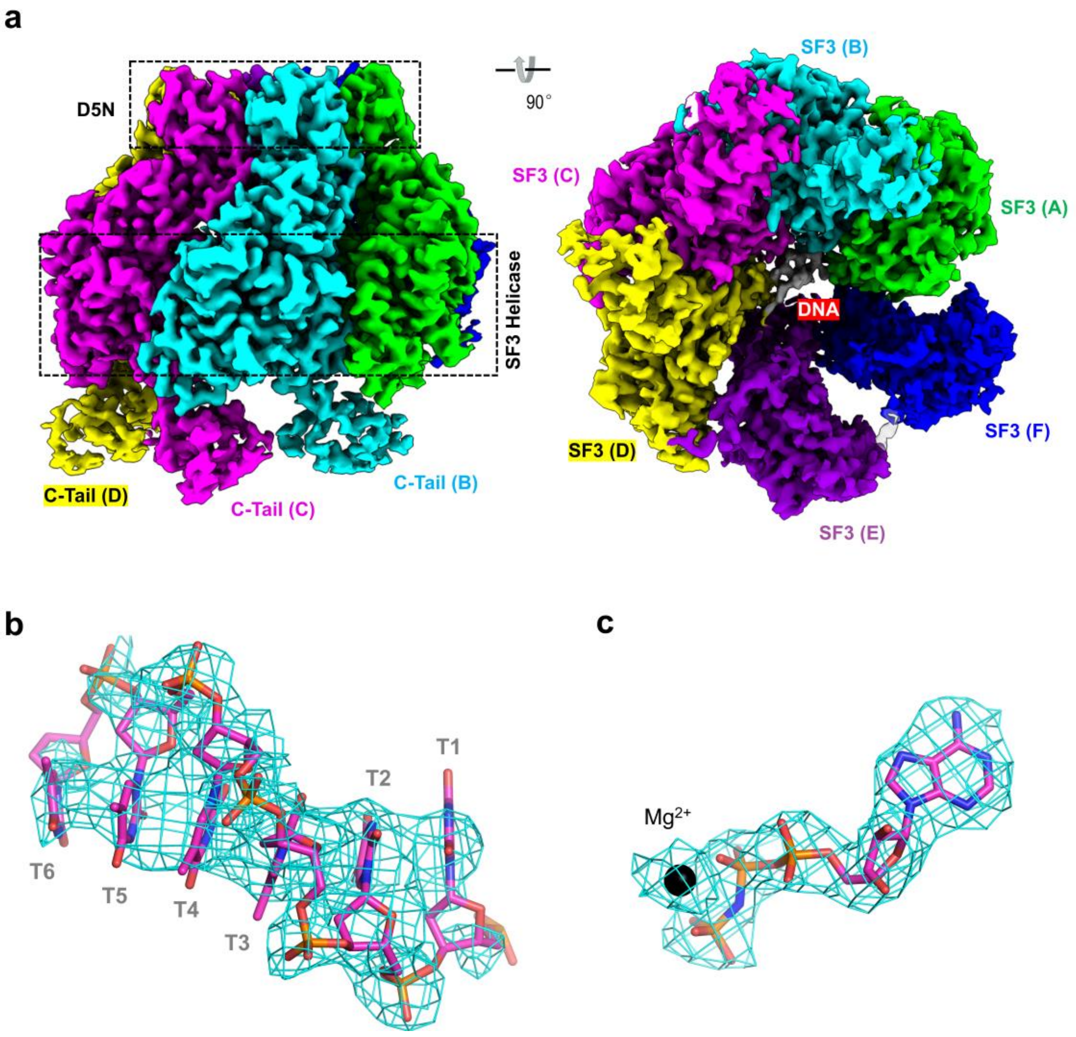

Supplementary Fig. S8 Electron density maps of the E5-ssDNA-AMPPNP structure. a The final electron density map of the MPXV E5/DNA complex. **b-c** Electron density map of the bound DNA and AMPPNP, respectively.

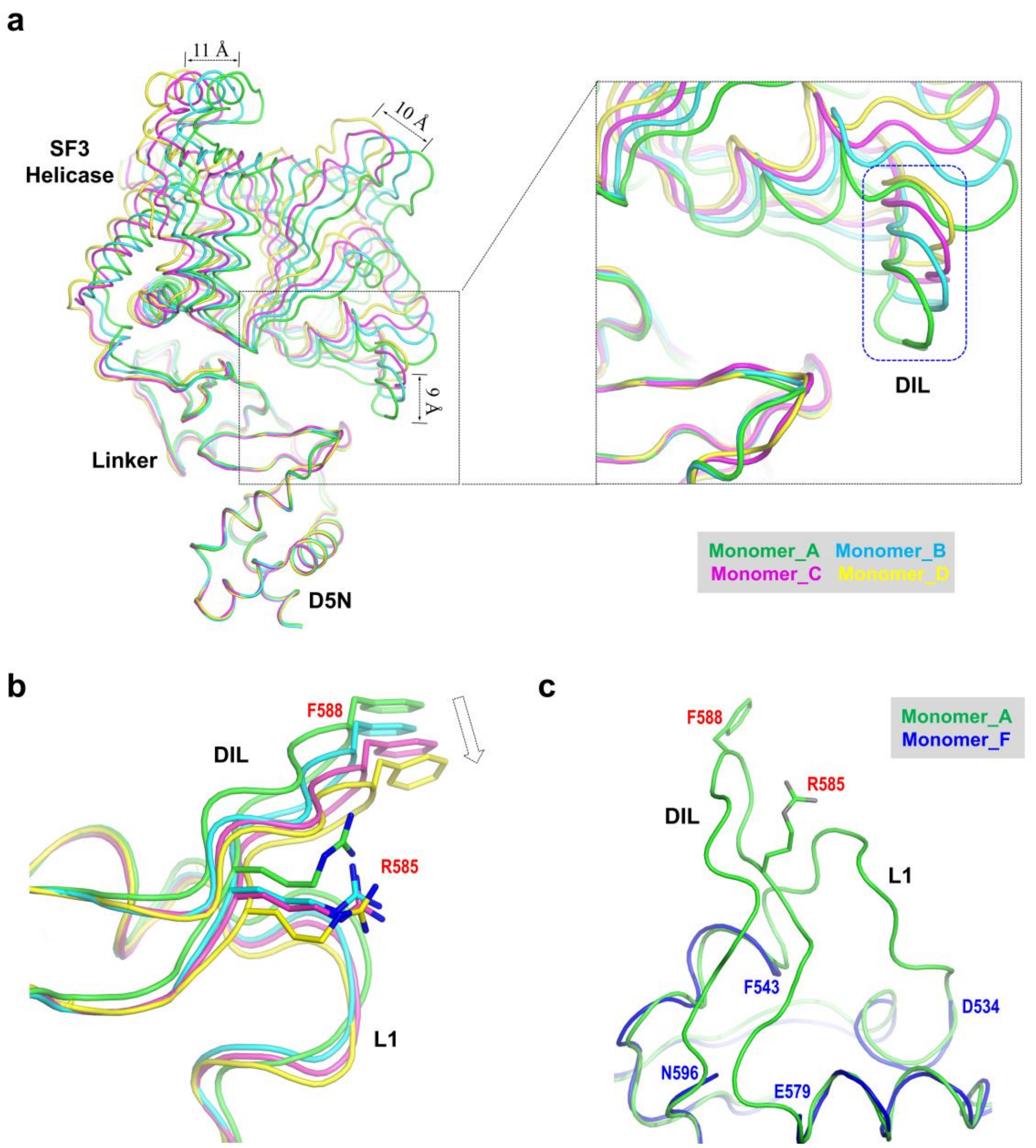

Supplementary Fig. S9 Conformational comparison of the SF3 domains in the E5-ssDNA-AMPPNP structure. a Superposition showing the tilting of the AEP domains. **b** Comparison of the DIL loops in monomers A to D. **c** Superposition showing the disordering of the DIL loop in monomer F.

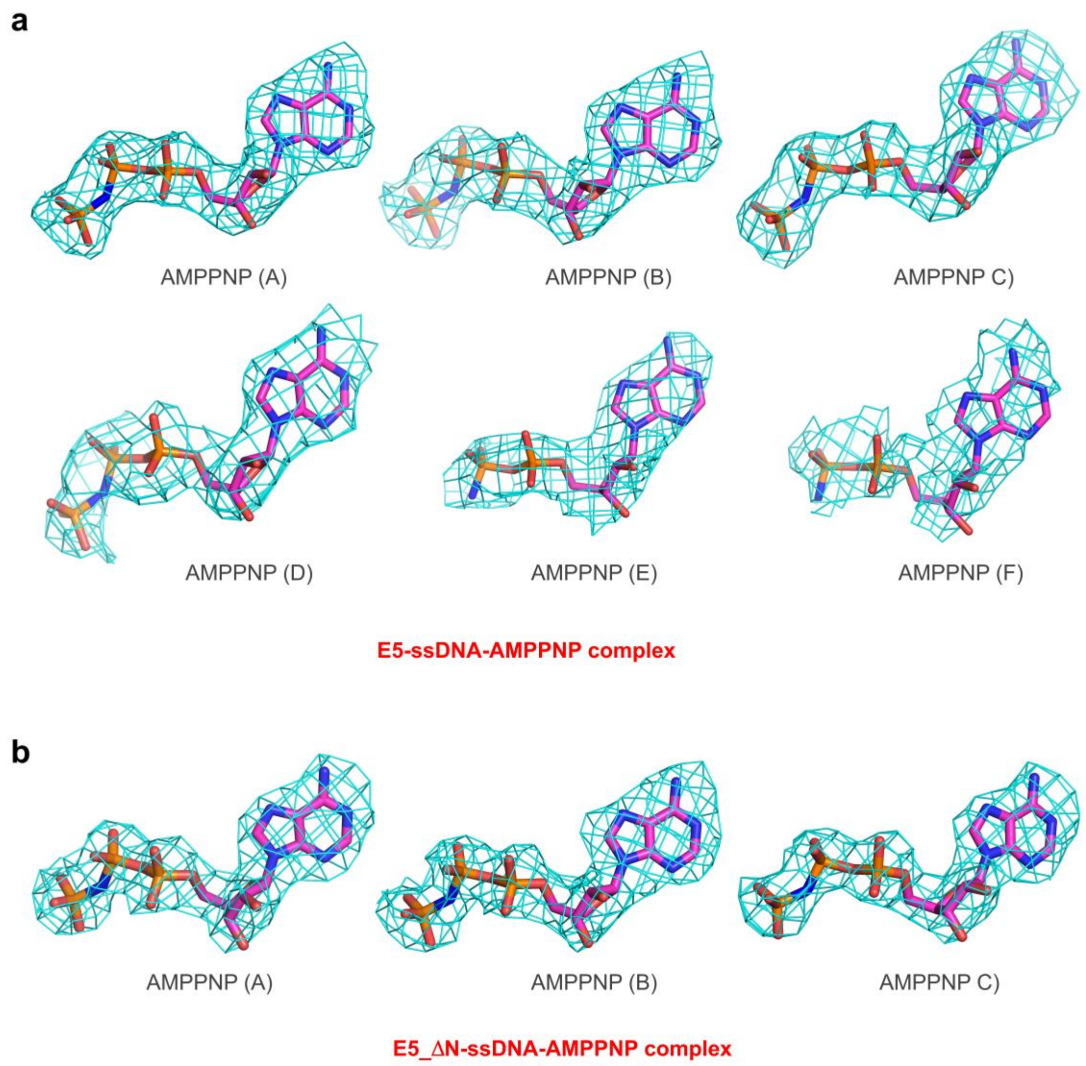

Supplementary Fig. S10 Electron density maps of AMPPNPs bound in the E5-ssDNA-AMPPNP structure and the E5_ΔN-ssDNA-AMPPNP structure.

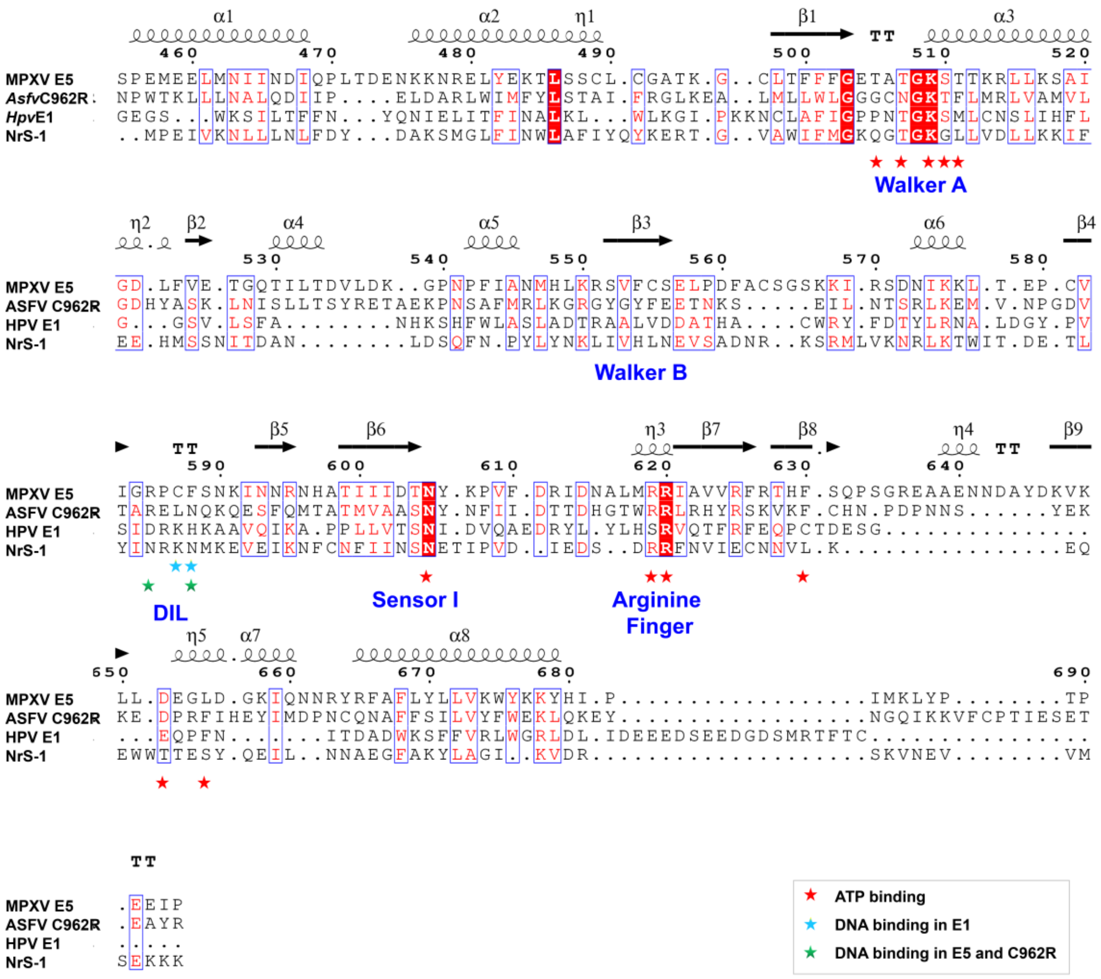

Supplementary Fig. S11 Sequence alignment of the SF3 helicase domains of MPXV E5 and the homologous proteins.

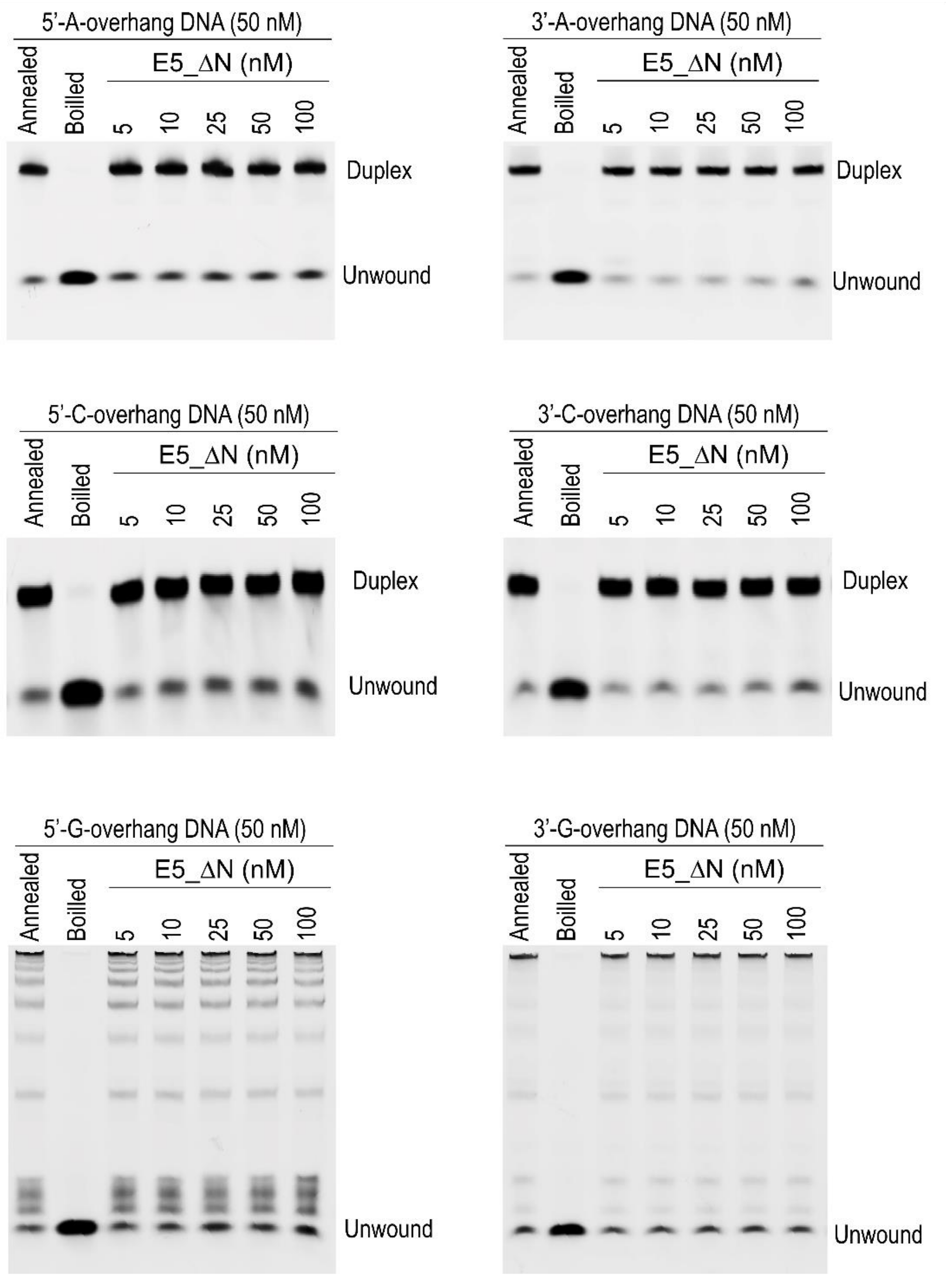

Supplementary Fig. S12 ***In vitro*** **DNA unwinding assays catalyzed by E5_ΔN protein**. Different from the other four overhang DNAs, the DNAs with G15 at their 5’ or 3’ ends showed multiple bands on the gel, which may be due to the formation of quadruplex structures. No DNA unwinding activity could be detected.

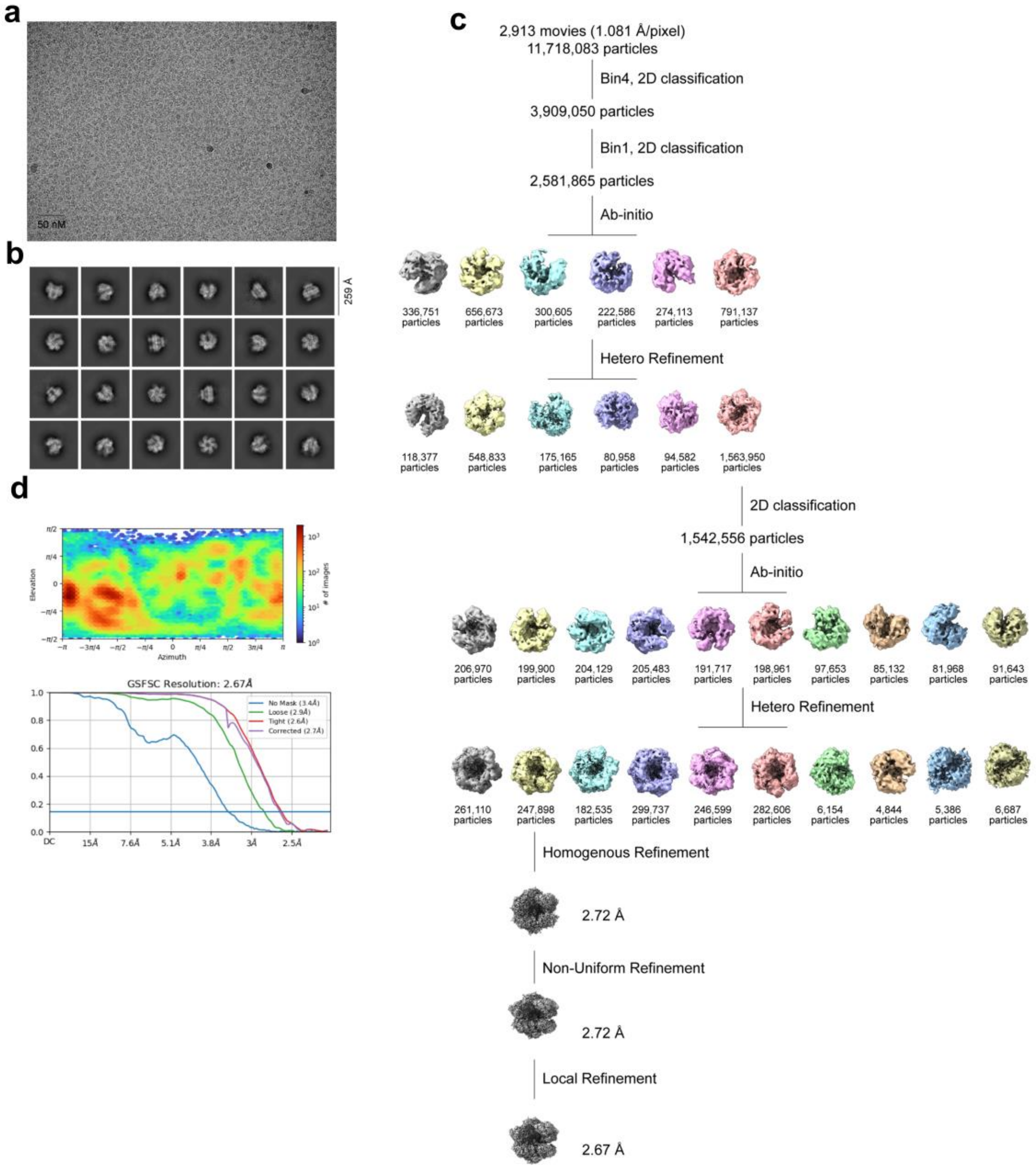

Supplementary Fig. S13 Cryo-EM data processing of the E5_ΔN-ssDNA-AMPPNP structure. a A representative cryo-EM image of the E5_ΔN-ssDNA-AMPPNP structure. **b** Representative 2D-classification images of the E5_ΔN-ssDNA-AMPPNP structure. **c** Workflow of cryo-EM data processing. **d** Particle distribution and Gold-standard Fourier shell correlation (GSFSC) of the final map of the E5_ΔN-ssDNA-AMPPNP structure.

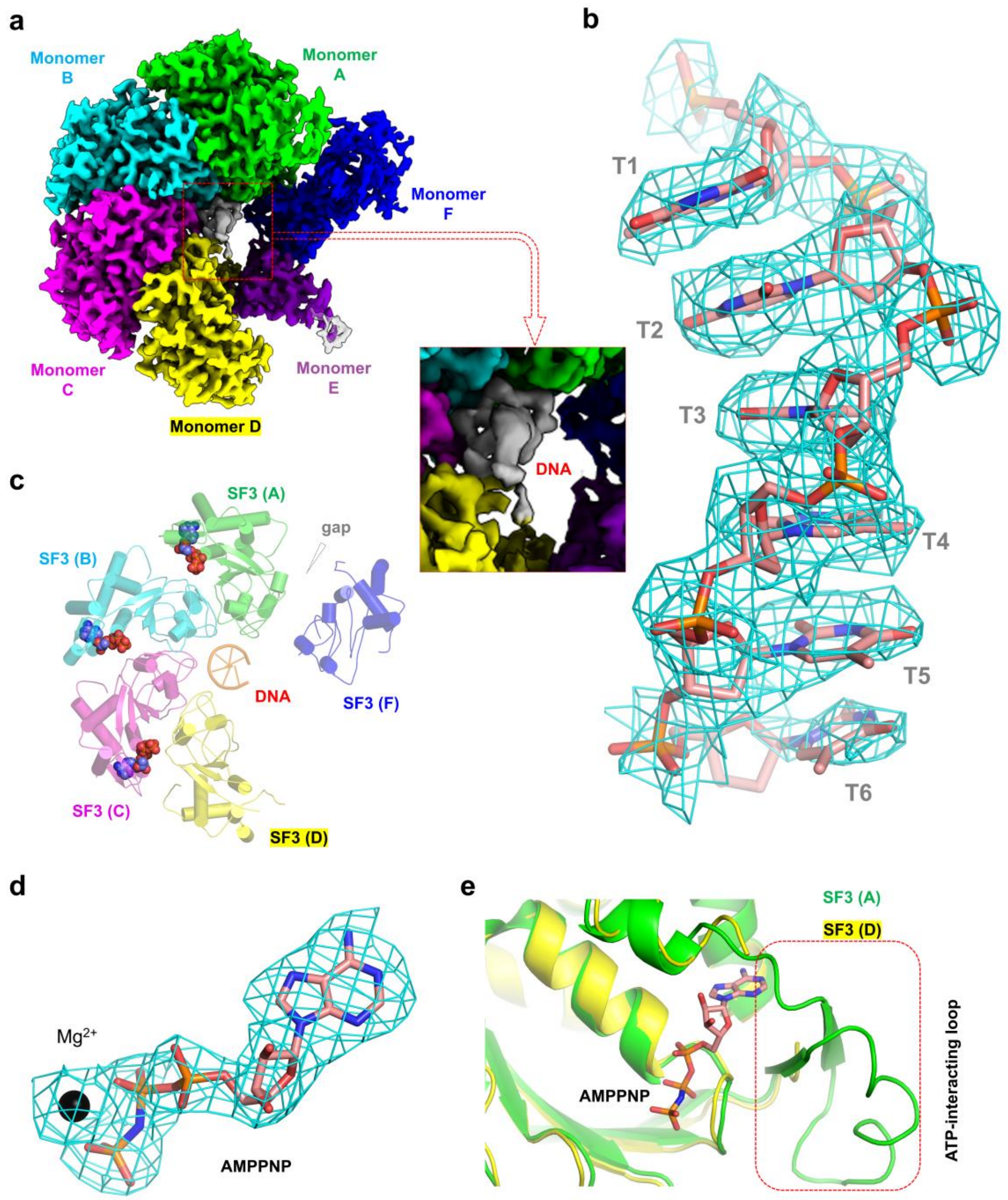

Supplementary Fig. S14 Electron density maps of the E5_ΔN-ssDNA-AMPPNP structure. a The final electron density map of the E5_ΔN-ssDNA-AMPPNP complex. **b** Electron density map of the bound DNA. **c** Conformation of the SF3 helicase domains, the bound DNA and AMPPNP in the E5_ΔN-ssDNA-AMPPNP complex. **d** Electron density map of the bound AMPPNP. **e** Superposition showing the disordering of the ATP-interacting loop in the monomer D, which is colored in yellow. Monomer A and the associated AMPPNP are color in green and atomic colors, respectively.

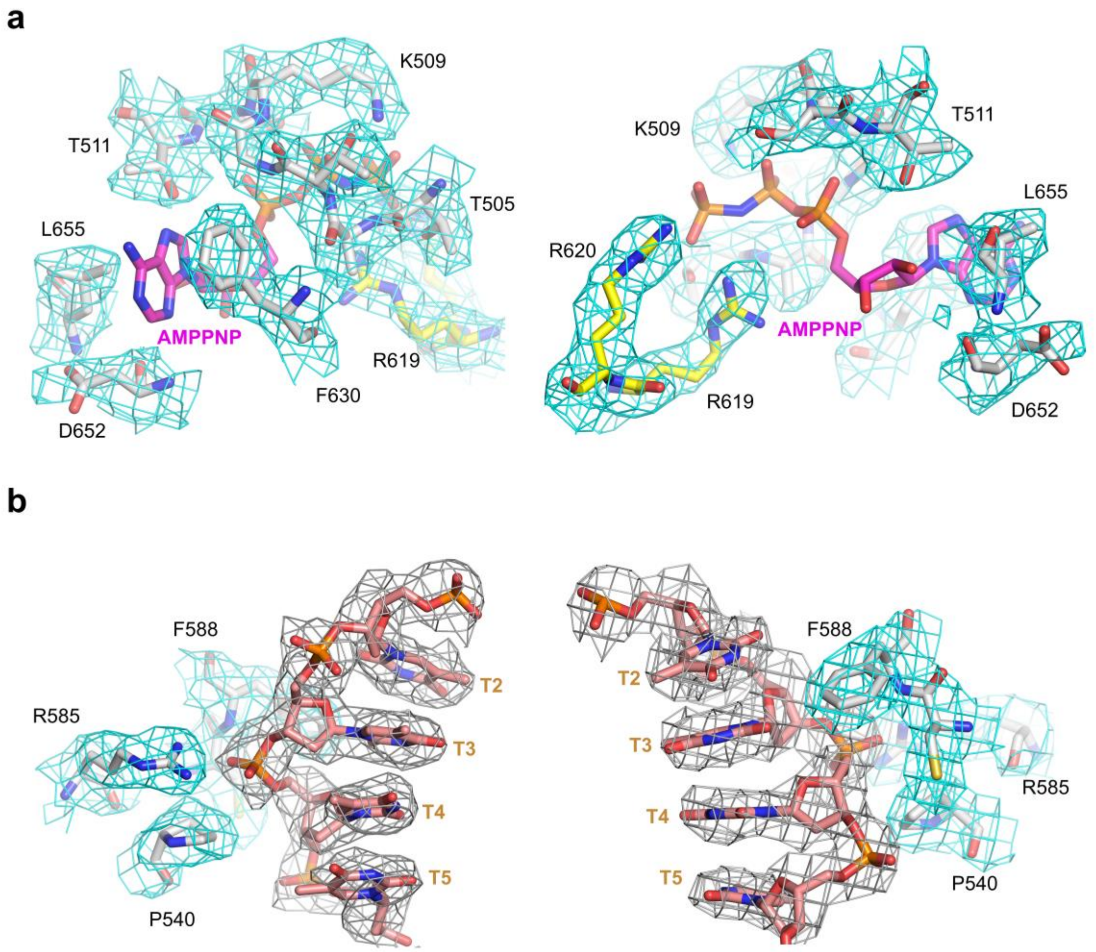

Supplementary Fig. S15 Electron density maps observed in the E5_ΔN-ssDNA-AMPPNP structure. a Electron density maps of the AMPPNP-binding residues. Electron density maps of AMPPNPs were omitted in this panel for clarity, but they can be found in Fig. S10B. **b** Electron density maps of the DNA-interacting residues.

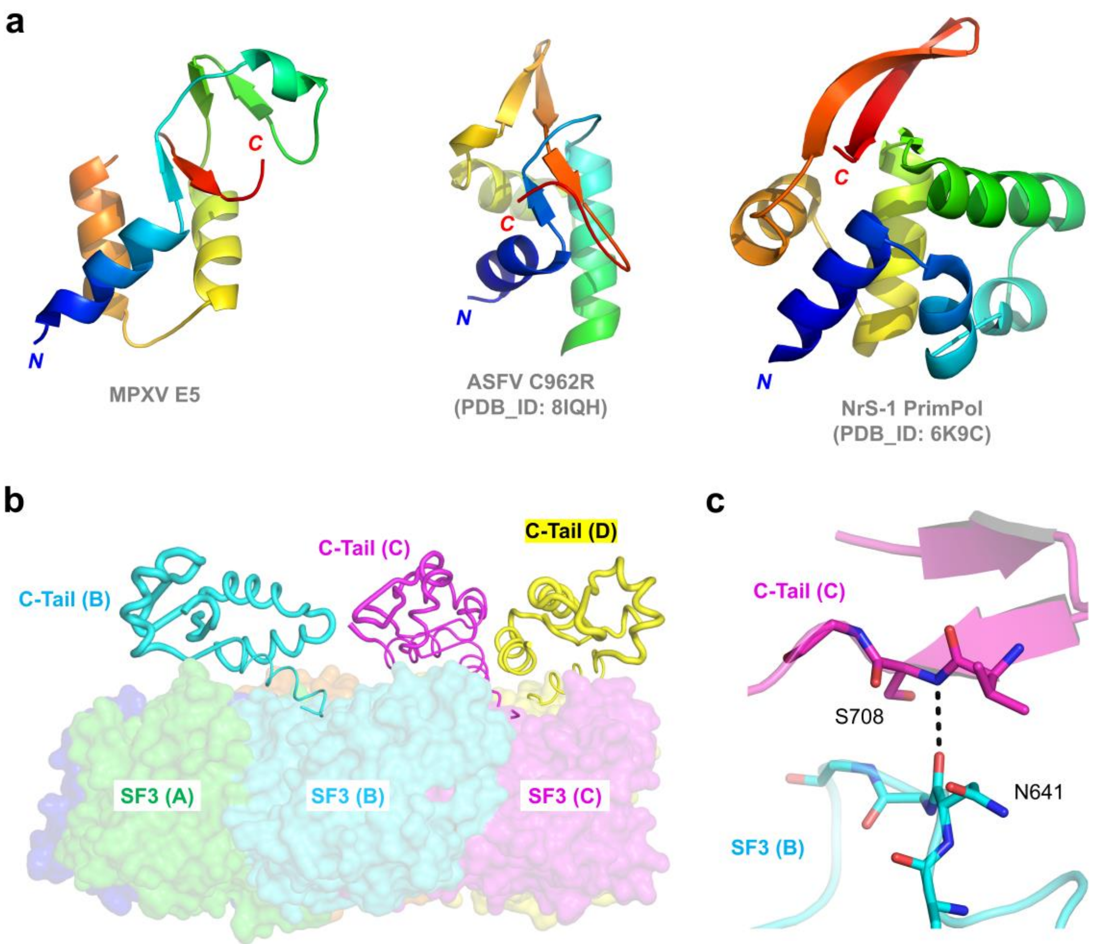

Supplementary Fig. S16 Folding and comparison of the C-Tail domains. a Overall folding and comparison of the C-Tail domain in MPXV E5 and homologous proteins. **b** Arrangement of the C-Tails in the E5-ssDNA-AMPPNP structure. **c** The H-bond interaction between the C-Tail and the AEP domain.

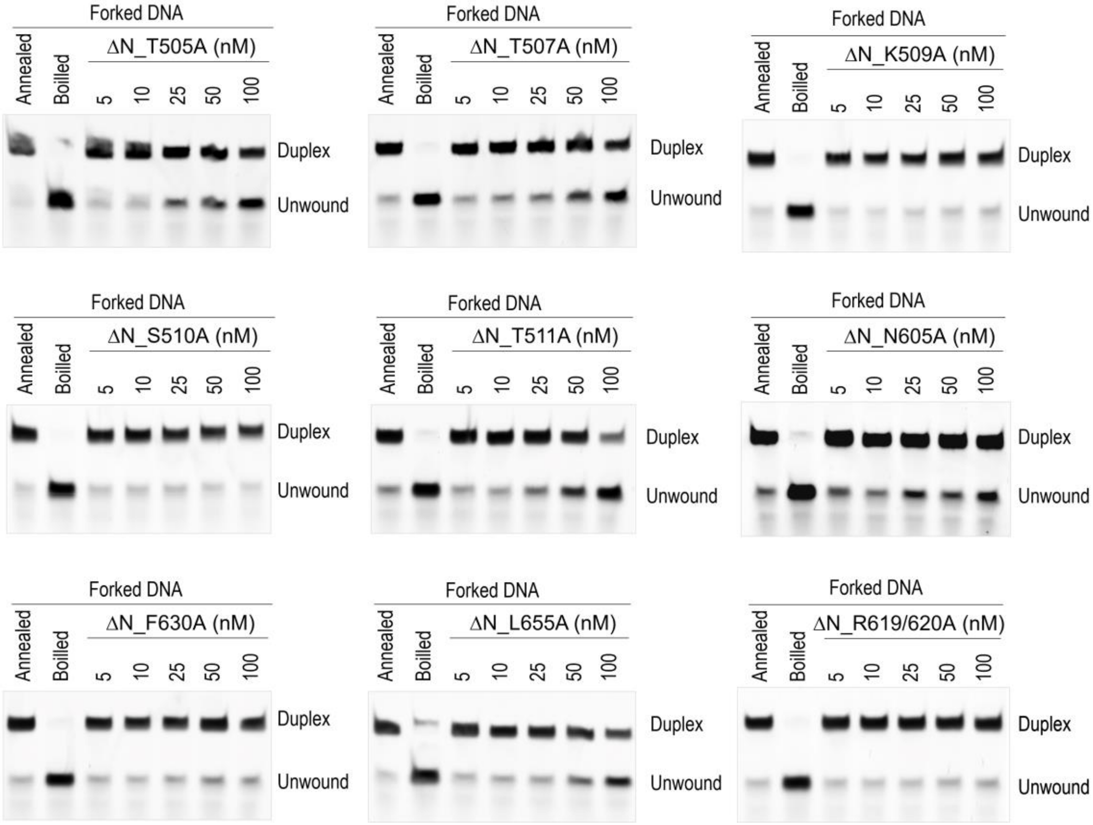

Supplementary Fig. S17 *In vitro* DNA unwinding assays catalyzed by mutated MPXV E5_ΔN proteins.

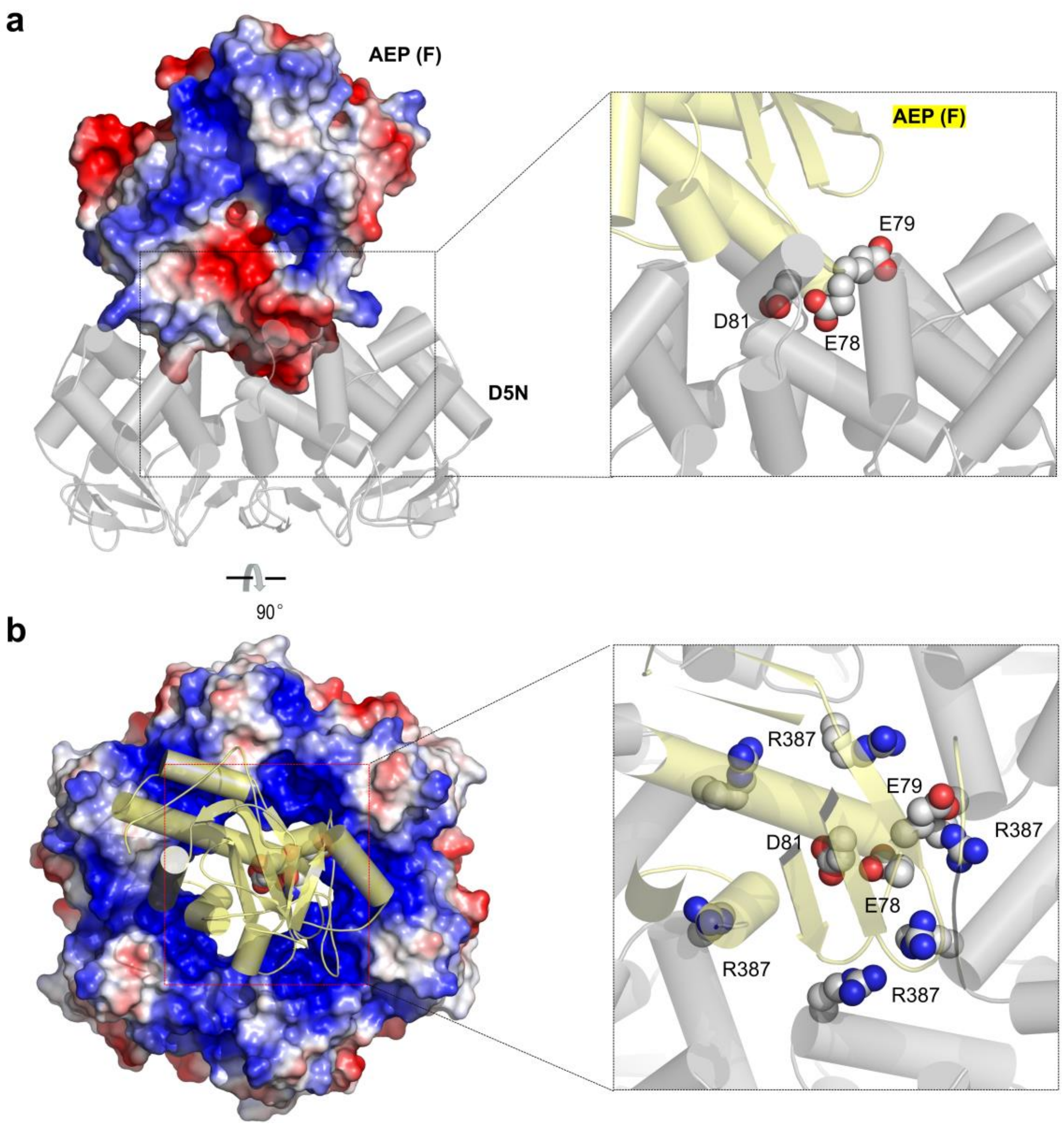

Supplementary Fig. S18 Analysis of the MPXV E5-AMPPNP structure. a Surface and cartoon presentation of the AEP domain. **b** Surface and cartoon presentation of the D5N domain. The positive and negatively charged residues are colored in blue and red on the surface presentation, respectively.

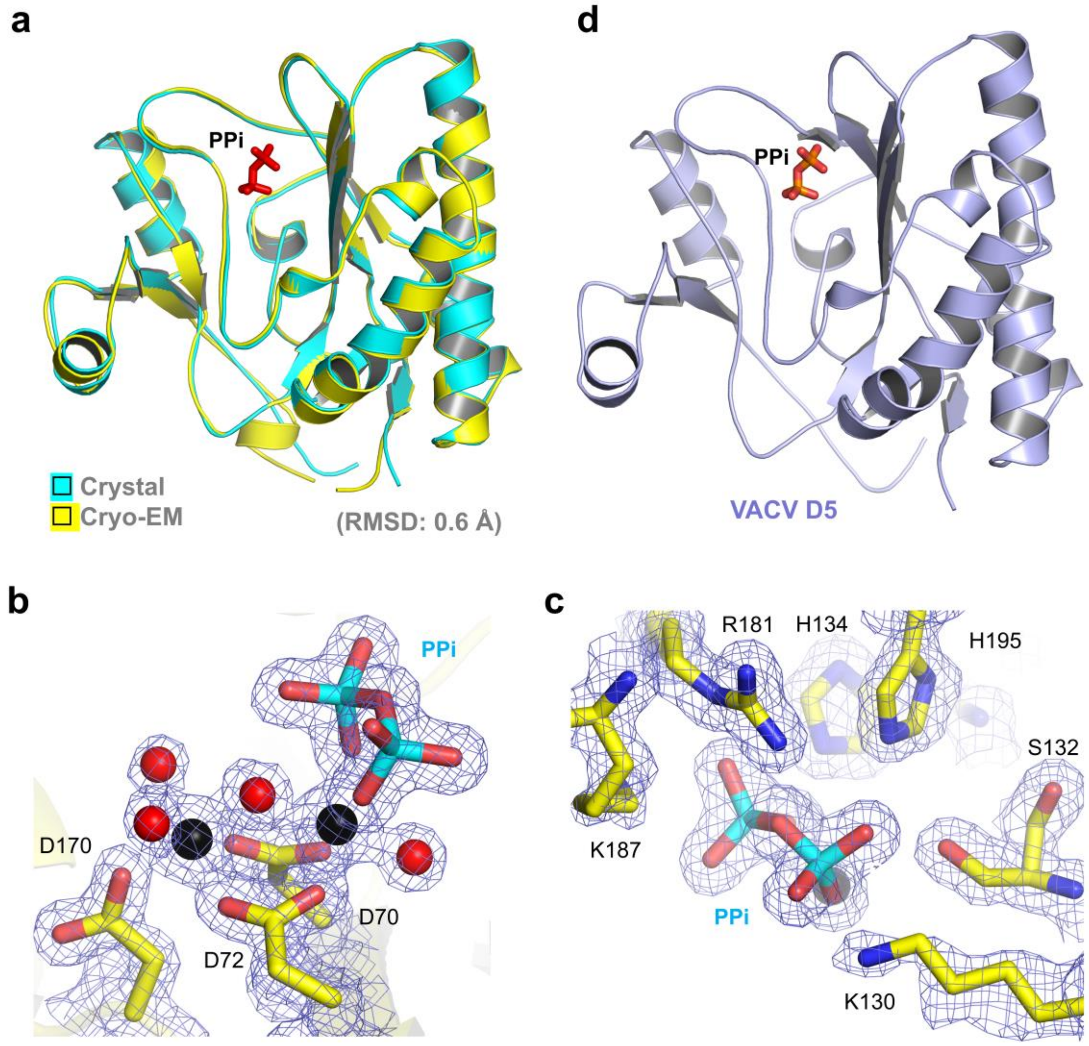

Supplementary Fig. S19 Structural analysis of the AEP domains. a Comparison of MPXV E5 AEP domains determined by cryo-EM and X-ray crystallographic methods. **b-c** The $2F_o$-F_c electron density maps of Mg^{2+}, PPi and the interacting residues in the MPXV E5 AEP structure. The map is contoured at 1.2 sigma level. **d** The overall folding of the VACV D5 AEP structure.

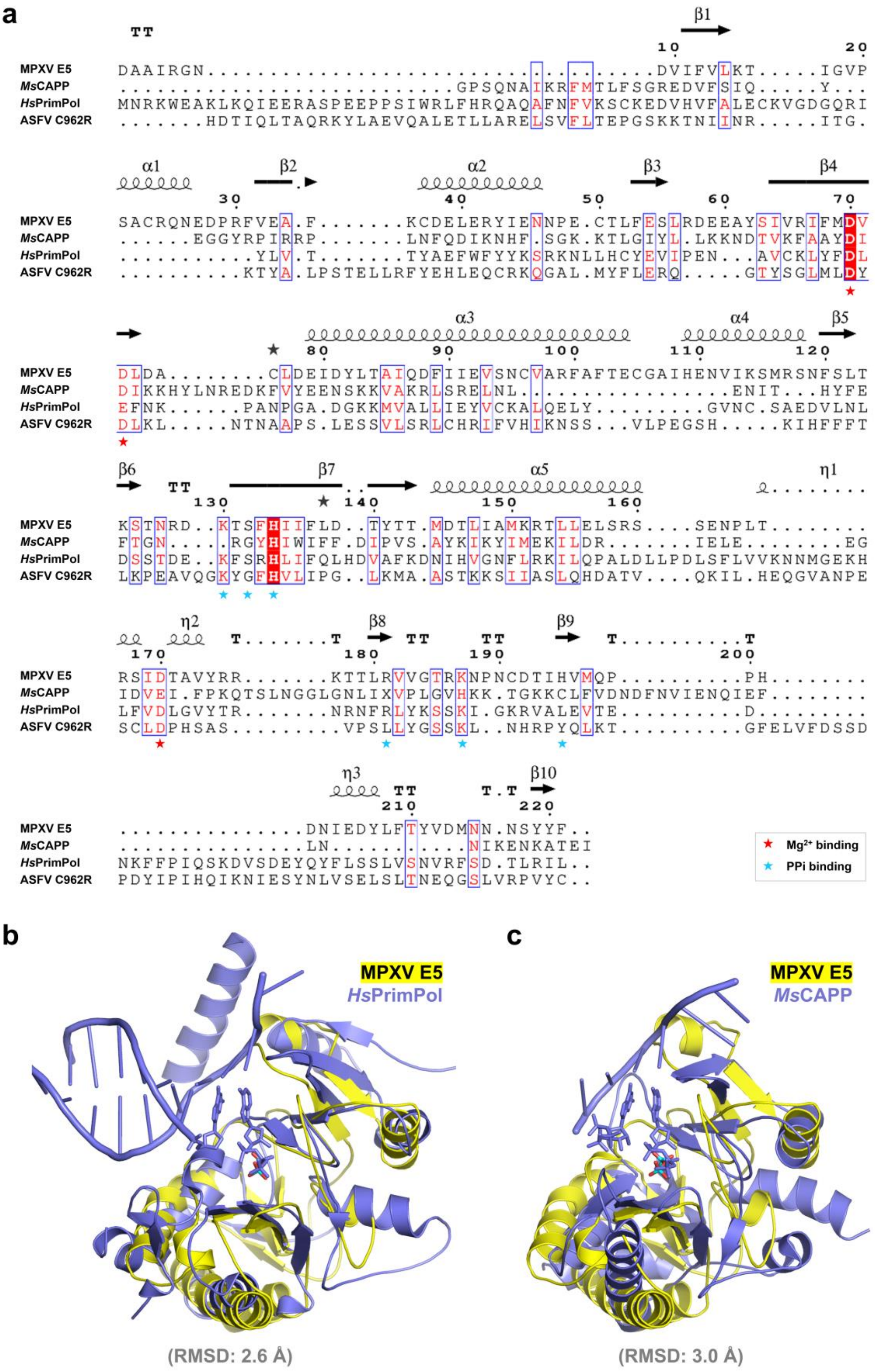

Supplementary Fig. S20 Comparison of the AEP domains. a Sequence alignment of the AEP domains of MPXV E5 and the homologous proteins. **b-c** Superposition of

MPXV E5 AEP domain structure with the *Hs*PrimPol/DNA complex and the *Ms*CAPP/DNA complex, respectively. MPXV E5 AEP domain is colored in yellow, whereas both *Hs*Prim and *Ms*CAPP structures are colored in light-blue.

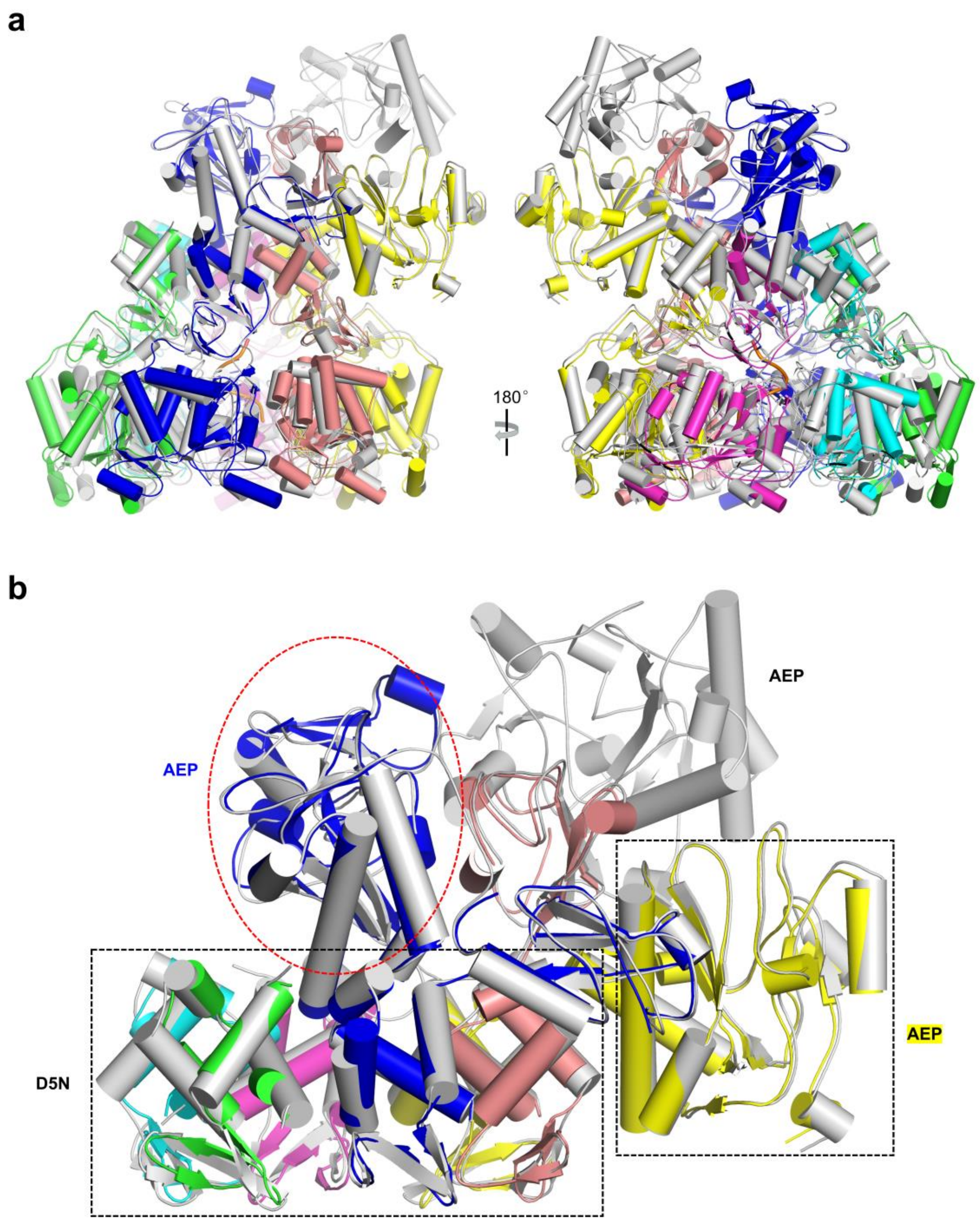

Supplementary Fig. S21 Comparison of our E5-AMPPNP structure and the reported 8HWA structure of MPXV helicase. All monomers in the 8HWA structure are colored in gray, whereas the monomers A-F in our E5-AMPPNP structure are shown in different colors.

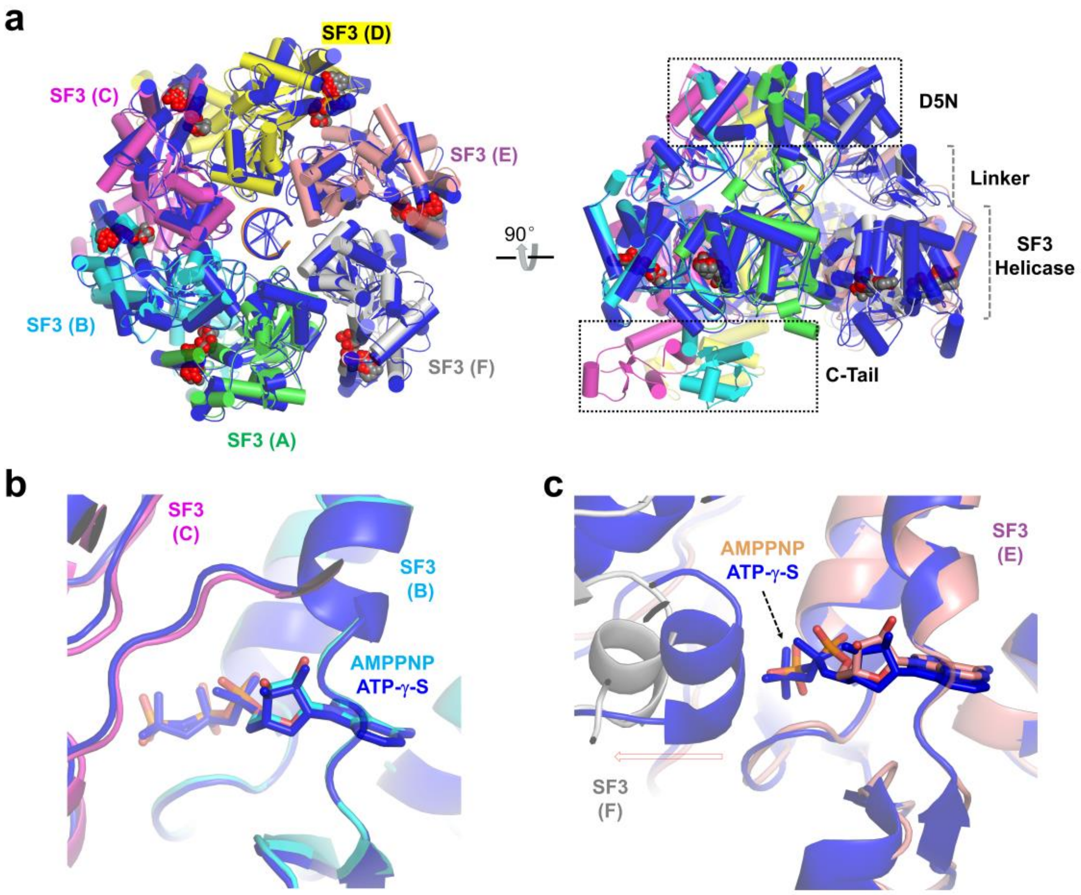

Supplementary Fig. S22 Comparison of our E5-ssDNA-AMPPNP structure and the reported 8HWG structure of MPXV helicase. The helicase monomers, the bound DNA and ATP-γ-S are colored in blue in the 8HWG structure, whereas they were shown in different colors in our E5-ssDNA-AMPPNP structure.

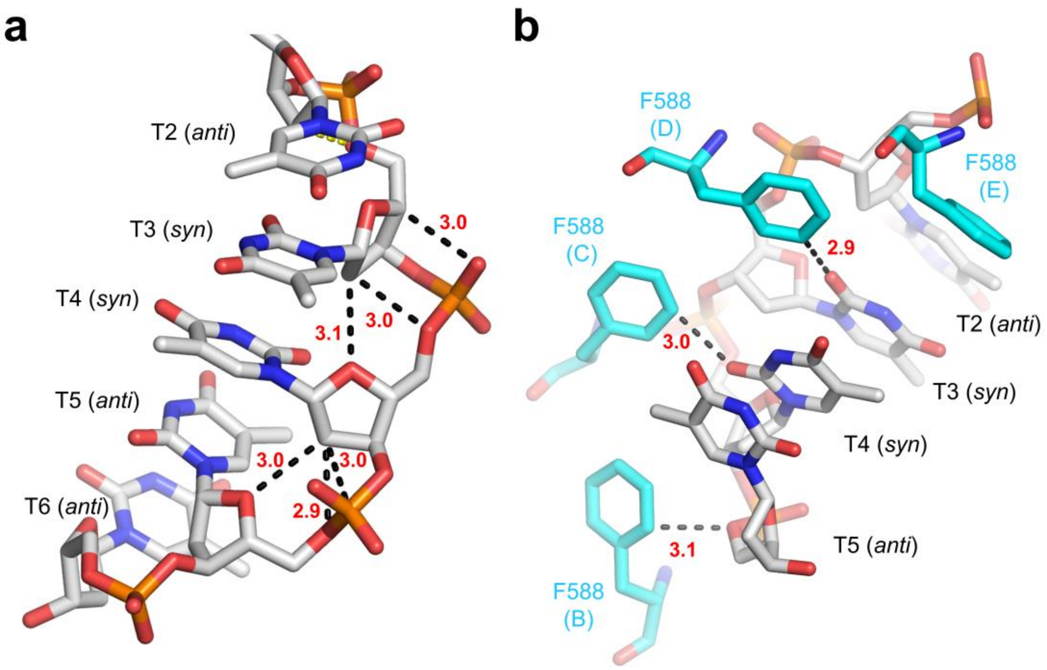

Supplementary Fig. S23 Close contacts observed in the reported 8HWG structure.

Close contacts are indicated by black dashed lines and numbers in red.

Supplementary Table S1 DNAs used in crystallization, cryo-EM and *in vitro* assays.

Name	Sequence（5'-3'）
5′-T-overhang DNA	Top strand: TTTTTTTTTTTTTTTCGGGATGCGCTTGACTCGTT
	Bottom strand: FAM-AACGAGTCAAGCGCATCCCG
5′-A-overhang DNA	Top strand: AAAAAAAAAAAAAAACGGGATGCGCTTGACTCGTT
	Bottom strand: FAM-AACGAGTCAAGCGCATCCCG
5′-C-overhang DNA	Top strand: CCCCCCCCCCCCCCCCGGGATGCGCTTGACTCGTT
	Bottom strand: FAM-AACGAGTCAAGCGCATCCCG
5′-G-overhang DNA	Top strand: GGGGGGGGGGGGGGGCGGGATGCGCTTGACTCGTT
	Bottom strand: FAM-AACGAGTCAAGCGCATCCCG
3′-T-overhang DNA	Top strand: AACGAGTCAAGCGCATCCCGTTTTTTTTTTTTTTT
	Bottom strand: FAM-CGGGATGCGCTTGACTCGTT
3′-A-overhang DNA	Top strand: AACGAGTCAAGCGCATCCCGAAAAAAAAAAAAAAA
	Bottom strand: FAM-CGGGATGCGCTTGACTCGTT
3′-C-overhang DNA	Top strand: AACGAGTCAAGCGCATCCCGCCCCCCCCCCCCCCC
	Bottom strand: FAM-CGGGATGCGCTTGACTCGTT
3′-G-overhang DNA	Top strand: AACGAGTCAAGCGCATCCCGGGGGGGGGGGGGGGG
	Bottom strand: FAM-CGGGATGCGCTTGACTCGTT
Forked DNA	Top strand: AACGAGTCAAGCGCATCCCGTTTTTTTTTTTTTTT
	Bottom strand: FAM-TTTTTTTTTTTTTTTCGGGATGCGCTTGACTCGTT
DNA/RNA	Template strand: TCAGTCAGTCTCTCTCTCTTCGGATTTTTTTTTT
	Primer strand: FAM-UCCGAA
Template ssDNA/NTPs	AAAAATCAA+GTP+ATP
	AAAAATCAAAA+GTP+ATP

Supplementary Table S2 Cryo-EM data collection, processing, model refinement and validation statistics

**Structure**	E5-AMPPNP	E5-ssDNA-AMPPNP	E5_ΔN-ssDNA-AMPPNP
PDB ID	8XJ6	8XJ7	8XJ8
EMDB ID	EMD-38394	EMD-38395	EMD-38396
**Data collection and Processing**			
Microscope Model	FEI TITAN KRIOS		
Detector Type	GATAN K3 (6k x 4k)		
Nominal CS	2.7		
Nominal Magnification	81000		
Pixel size (Å)	1.0773		1.081
Electron dose (e^-/$Å^2$)	50		
Defocus range (μm)	-1.0 to -2.0		
Micrograph Number	5239		2913
**Reconstruction**			
Software	cryoSPARC		
Particles picked	1,185,068		2,581,865
Particles refinement	126,172	221,856	247,898
Symmetry	C1	C1	C1
Resolution (Å)	3.32	2.74	2.67
**Refinement**			
Software	Phenix		
Model Composition			
Number of atoms	22688	19595	15904
Protein residues	2807	2420	1957
Nucleotides	0	6	6
Ligand (ZN^{2+}/Mg^{2+}/ANP)	2/0/6	0/3/6	0/3/3
Bonds RMSD			
Bonds lengths (Å)	0.004	0.003	0.003
Bonds angels (°)	0.796	0.590	0.640
**Validation**			
MolProbity score	1.91	1.98	1.86
Clash score	12.83	8.63	5.62
Rotamer outliers (%)	0.40	1.55	2.00
C-beta outliers (%)	0.00	0.04	0.00
Ramachandran plot			
Favored (%)	95.84	94.63	95.28
Allowed (%)	3.56	5.08	4.00
Outlier (%)	0.51	0.29	0.73
Model vs. Data			
CC mask	0.78	0.84	0.85
CC volume	0.77	0.82	0.83

Supplementary Table S3: Crystal data collection and refinement statistics.

Structure	VACV D5-AEP	MPXV E5-AEP
PDB ID	8XIF	8XIG
Data collection a		
Space group	$P2_12_12_1$	$P2_12_12_1$
Cell parameter:		
a, b, c (Å)	52.47, 56.62, 74.16	51.98, 54.82, 74.85
α, β, γ (°)	90.0, 90.0, 90.0	90.0, 90.0, 90.0
Wavelength (Å)	0.97	0.97
Resolution (Å)	28.31-1.39	30.00-1.65
High-resolution shell (Å)	1.43-1.39	1.71-1.65
Completeness (%)	100.0(100.0)	100.0(91.9)
Redundancy	12.8(13.0)	10.2(6.9)
Rmerge (%)	9.8(194.5)	11.7(49.1)
I/σ(I)	13.8(1.6)	23.1(1.4)
Refinement		
Resolution (Å)	28.31-1.39	27.41-1.65
No. of reflections	45138	23492
Rwork (%) / Rfree (%)	17.91/19.72	17.31/21.63
No. of atoms		
Protein	1803	1786
PPV	1	1
Mg	2	2
Water	172	361
R.m.s. deviations		
Bond length (Å)	0.008	0.007
Bond angle (°)	1.048	0.970
Ramachandran plot (%)		
Most favorable	98.19	97.75
Additional allowed	1.81	2.25
Outlier	0.00	0.00

a: Values in parentheses are for the high-resolution shell.

Supplementary Table S4 Coden-optimized cDNA sequences of MPXV E5 and VACV D5

The optimized cDNA sequence of MPXV E5 (from 5' to 3') [a]

GGATCCATGGATGCGGCGATCCGTGGTAACGACGTTATCTTCGTACTTAAGACCATCGGTGTTCCATCTGCATGT
CGTCAGAACGAAGATCCGCGTTTCGTGGAAGCGTTCAAATGCGACGAACTGGAACGTTACATCGACAACAATCCG
GAATGCACCTTGTTCGAAAGCCTGCGTGACGAAGAGGCTTACTCTATCGTTCGTATCTTCATGGATGTTGACCTG
GATGCATGCCTGGACGAAATCGACTACCTGACCGCAATCCAAGACTTCATCATCGAAGTTTCTAACTGCGTTGCT
CGTTTCGCATTCACCGAATGCGGTGCGATCCACGAGAACGTGATCAAATCCATGCGTTCTAACTTCTCTCTGACC
AAATCTACCAACCGTGACAAGACCTCTTTCCACATCATCTTTCTGGACACTTACACCACTATGGACACTCTGATC
GCGATGAAACGTACTCTGCTGGAACTGTCTCGTTCTAGCGAGAATCCACTGACTCGTTCTATCGACACTGCGGTT
TACCGTCGTAAGACCACTCTGCGTGTTGTTGGTACTCGTAAGAATCCAAACTGCGACACCATCCACGTGATGCAG
CCGCCGCATGACAACATCGAAGATTACCTGTTCACCTACGTTGACATGAACAACAACAGCTACTACTTCAGCCTG
CAGCGTCGTCTCGAAGACCTGGTTCCGGATAAACTGTGGGAACCGGGCTTCATCTCTTTCGAAGACGCGATCAAA
CGTGTTTCTAAGATCTTCATCAACTCTATCATCAACTTCAACGACCTGGACGAGAACAACTTCACCACCGTTCCG
CTGGTTATCGACTACGTTACTCCATGTGCTCTGTGCAAGAAACGTAGCCACAAACACCCACACCAGCTGTCTCTG
GAAAACGGTGCTATCCGTATCTACAAGACCGGTAATCCGCACTCTTGCAAAGTTAAGATCGTTCCGCTGGACGGT
AACAAACTGTTCAACATCGCTCAGCGTATTCTGGACACCAACTCCGTTCTGCTGACCGAACGTGGTGACCACATC
GTTTGGATCAACAACTCTTGGAAGTTCAACTCTGAAGAACCGCTGATCACCAAACTGATTCTGTCTATCCGTCAC
CAGCTTCCGAAAGAATACTCTTCTGAACTGCTGTGTCCGCGTAAACGTAAAACCGTTGAAGCGAACATCCGTGAC
ATGCTGGTTGACTCTGTGGAAACCGACACCTATCCGGACAAACTGCCGTTCAAGAACGGTGTGCTGGATCTGGTT
GACGGTATGTTCTACTCTGGTGATGACGCGAAGAAATACACTTGCACCGTTTCTACCGGTTTCAAATTCGACGAC
ACCAAATTCGTTGAAGACAGTCCGGAAATGGAAGAACTGATGAACATCATCAACGACATCCAGCCGCTGACCGAT
GAGAACAAGAAGAACCGTGAACTGTACGAGAAGACTCTGTCTAGCTGCCTGTGCGGTGCAACCAAAGGTTGCCTG
ACCTTCTTCTTCGGTGAAACTGCGACTGGTAAATCTACCACCAAACGTCTGCTGAAATCTGCTATCGGTGACCTG
TTCGTTGAAACCGGTCAGACCATCTTGACCGACGTTCTGGACAAAGGTCCAAATCCATTCATCGCTAACATGCAC
CTGAAACGTTCTGTGTTCTGCTCTGAATTGCCGGATTTCGCGTGCTCCGGTAGCAAGAAGATCCGTTCTGACAAC
ATCAAGAAACTGACTGAACCATGCGTTATCGGTCGTCCGTGCTTCTCCAACAAGATCAACAACCGTAACCACGCT
ACCATCATCATCGACACTAACTACAAACCGGTGTTCGACCGTATCGACAACGCGCTGATGCGTCGTATCGCTGTA
GTTCGTTTCCGTACTCACTTCAGCCAGCCGTCTGGTCGTGAAGCAGCGGAGAACAACGATGCGTACGACAAAGTT
AAACTGCTGGACGAAGGTCTGGATGGTAAGATCCAGAACAACCGTTACCGTTTCGCGTTTCTGTACCTGCTGGTT
AAATGGTACAAGAAGTACCACATTCCGATCATGAAACTGTATCCGACTCCGGAAGAAATTCCAGACTTCGCGTTC
TACCTGAAGATCGGCACCTTGCTGGTGTCTTCTTCCGTTAAACACATTCCGCTGATGACCGATCTGTCTAAGAAA
GGTTACATCCTGTACGACAACGTTGTTACCTTGCCACTGACCACCTTCCAGCAGAAGATCTCCAAATACTTCAAC
TCTCGTCTGTTCGGTCACGACATCGAGAGCTTCATCAACCGTCATAAAAAGTTCGCAAACGTTTCTGACGAATAC
CTGCAGTACATCTTCATCGAAGACATCTCTAGCCCGTAACTCGAG

The optimized cDNA sequence of VACV D5 (from 5' to 3')

GGATCCATGGATGCCGCAATTCGTGGTAATGATGTTATTTTCGTTCTGAAAACCATCGGTGTTCCGAGCGCCTGT
CGTCAGAACGAAGATCCGCGCTTTGTTGAAGCATTTAAATGTGATGAACTGGAACGTTATATCGAAAATAATCCG
GAATGTACCCTGTTTGAAAGCCTGCGTGATGAAGAAGCATATAGCATTGTGCGTATTTTCATGGATGTGGATCTG
GATGCATGTCTGGATGAAATTGATTATCTGACGGCAATCCAGGATTTTATTATTGAAGTTAGCAACTGTGTGGCA
CGTTTTGCGTTTACGGAATGTGGTGCAATTCATGAAAATGTTATTAAAAGCATGCGCAGTAATTTTAGCCTGACC
AAAAGCACCAATCGTGATAAAACAAGTTTTCATATCATCTTTCTGGATACATATACCACCATGGATACCCTGATT
GCAATGAAACGCACCCTGCTGGAACTGAGCCGTTCAAGCGAAAATCCGCTGACCCGTAGCATTGATACCGCCGTT

```
TATCGTCGTAAAACCACCCTGCGCGTTGTTGGTACCCGTAAAAATCCGAATTGTGATACCATTCATGTTATGCAG
CCGCCGCATGATAATATTGAAGATTATCTGTTTACATATGTTGATATGAACAACAACTCCTATTATTTTAGCCTG
CAGCAGCGTCTGGAAGATCTGGTGCCGGATAAACTGTGGGAACCTGGTTTTATTAGCTTTGAAGATGCCATTAAA
CGTGTTAGCAAAATTTTCATTAACTCCATTATCAACTTTAACGATCTGGATGAAAACAATTTTACCACCGTTCCG
CTGGTTATTGATTATGTTACCCCGTGCGCACTGTGTAAGAAGCGTAGTCATAAACATCCGCATCAGCTGAGCCTG
GAAAATGGCGCAATTCGTATTTATAAAACCGGCAATCCGCATAGCTGTAAAGTTAAAATTGTTCCTCTGGATGGT
TAACTCGAG
```

[a]: ***GGATCC*** and ***CTCGAG*** at the 5'-end and 3'-end are Bam HI and Xho I recognition sequence.

Supplementary Table S5 Primers used for mutant or truncated MPXV E5 and VACV D5 construction.

Name	Sequence (5'-3')
D5-SUMO_F	AGAGAACAGATTGGTGGATCCATGGATGCCGCAATTCG
D5_1-322-SUMO_R	GTGGTGGTGGTGGTGCTCGAGTTAATCCAGAGGAACAATTTTAAC
D5_1-230-SUMO_R	GTGGTGGTGGTGGTGCTCGAGTTACAGATCTTCCAGACGCTGC
E5-SUMO_F	AGAGAACAGATTGGTGGATCCATGGATGCGGCGATCCG
E5-SUMO_R	GTGGTGGTGGTGGTGCTCGAGTTACGGGCTAGAGATGTCTTCGATG
E5_1-322-SUMO_R	GTGGTGGTGGTGGTGCTCGAGTTAGTCCAGCGGAACGATCTTAACTTTG
E5_323-785-SUMO_F	AGAGAACAGATTGGT GGATCCGGTAACAAACTGTTCAACATCGCTC
E5-Triple mutant_F	GATTGCGGTCAGGTAAGCGATTGCCGCCAG
E5-Triple mutant_R	CTGGATGCATGCCTGGCGGCAATCGCTTACCTGAC
E5_323-694-SUMO_R	GTGGTGGTGGTGGTGCTCGAGTTATGGAATTTCTTCCGGAGTCGG
E5_1-230-SUMO_R	GTGGTGGTGGTGGTGCTCGAGTTACAGGTCTTCGAGACGACGC
E5_T505A_F	TTCTTCTTCGGTGAAGCTGCGACTGGTAAATCT
E5_T505A_R	AGATTTACCAGTCGCAGCTTCACCGAAGAAGAA
E5_T507A_F	TTCGGTGAAACTGCGGCTGGTAAATCTACCACC
E5_T507A_R	GGTGGTAGATTTACCAGCCGCAGTTTCACCGAA
E5_N605A_F	ATCATCATCGACACTGCTTACAAACCGGTGTTC
E5_N605A_R	GAACACCGGTTTGTAAGCAGTGTCGATGATGAT
E5_K509A_F	TTCGGCTTGGGCAATCACGCCTTTCTTGAAGCTATC
E5_K509A_R	ACGTTTGGTGGTAGAAGCACCAGTCGCAGTTTC
E5_S510A_F	GAAACTGCGACTGGTAAAGCTACCACCAAACGT
E5_S510A_R	ACGTTTGGTGGTAGCTTTACCAGTCGCAGTTTC
E5_T511A_F	GAAACTGCGACTGGTAAATCTGCGACCAAACGT
E5_T511A_R	ACGTTTGGTCGCAGATTTACCAGTCGCAGTTTC
E5_F630A_F	CGTTTCCGTACTCACGCTAGCCAGCCGTCTGGT
E5_F630A_R	ACCAGACGGCTGGCTAGCGTGAGTACGGAAACG
E5_L655A_F	CTGCTGGACGAAGGTGCGGATGGTAAGATCCAG
E5_L655A_R	CTGGATCTTACCATCCGCACCTTCGTCCAGCAG
E5_R619A/R620A_F	GACAACGCGCTGATGGCGGCTATCGCTGTAGTTCGT
E5_R619A/R620A_R	ACGAACTACAGCGATAGCCGCCATCAGCGCGTTGTC
E5_R585A_F	CCATGCGTTATCGGTGCTCCGTGCTTCTCCAAC
E5_R585A_R	GTTGGAGAAGCACGGAGCACCGATAACGCATGG
E5_F588A_F	ATCGGTCGTCCGTGCGCTTCCAACAAGATCAAC
E5_F588A_R	GTTGATCTTGTTGGAAGCGCACGGACGACCGAT
E5_R387A_F	GAACTGCTGTGTCCGGCTAAACGTAAAACCGTT
E5_R387A_R	AACGGTTTTACGTTTAGCCGGACACAGCAGTTC
E5_D70A_F	GTTCGTATCTTCATGGCTGTTGACCTGGATGCA
E5_D70A_R	TGCATCCAGGTCAACAGCCATGAAGATACGAAC
E5_D72A_F	ATCTTCATGGATGTTGCACTGGATGCATGCCTG
E5_D72A_R	CAGGCATGCATCCAGTGCAACATCCATGAAGAT
E5_D170A_F	CTGACTCGTTCTATCGCAACTGCGGTTTACCGT
E5_D170A_R	ACGGTAAACCGCAGTTGCGATAGAACGAGTCAG

E5_K130A_F	TCTACCAACCGTGACGCGACCTCTTTCCACATC
E5_K130A_R	GATGTGGAAAGAGGTCGCGTCACGGTTGGTAGA
E5_S132A_F	AACCGTGACAAGACCGCTTTCCACATCATCTTT
E5_S132A_R	AAAGATGATGTGGAAAGCGGTCTTGTCACGGTT
E5_K187A_F	GTTGTTGGTACTCGTGCGAATCCAAACTGCGAC
E5_K187A_R	GTCGCAGTTTGGATTCGCACGAGTACCAACAAC
E5_R181A_F	CGTAAGACCACTCTGGCTGTTGTTGGTACTCGT
E5_R181A_R	ACGAGTACCAACAACAGCCAGAGTGGTCTTACG
E5_H195A_F	AACTGCGACACCATCGCTGTGATGCAGCCGCCG
E5_H195A_R	CGGCGGCTGCATCACAGCGATGGTGTCGCAGTT
E5_H134A_F	GACAAGACCTCTTTCGCAATCATCTTTCTGGAC
E5_H134A_R	GTCCAGAAAGATGATTGCGAAAGAGGTCTTGTC
